# The protective effect of inflammatory monocytes during systemic *C*. *albicans* infection is dependent on collaboration between C-type lectin-like receptors

**DOI:** 10.1371/journal.ppat.1007850

**Published:** 2019-06-26

**Authors:** Aiysha Thompson, Luke C. Davies, Chia-Te Liao, Diogo M. da Fonseca, James S. Griffiths, Robert Andrews, Adam V. Jones, Mathew Clement, Gordon D. Brown, Ian R. Humphreys, Philip R. Taylor, Selinda J. Orr

**Affiliations:** 1 Division of Infection and Immunity and Systems Immunity Research Institute, Cardiff University School of Medicine, Cardiff, Wales; 2 UK Dementia Research Institute at Cardiff, Cardiff, Wales; 3 University Dental Hospital, Cardiff and Vale University Health Board, Cardiff, Wales United Kingdom; 4 Medical Research Council Centre for Medical Mycology at the University of Aberdeen, Aberdeen Fungal Group, University of Aberdeen, Foresterhill, Aberdeen, United Kingdom; Memorial Sloan-Kettering Cancer Center, UNITED STATES

## Abstract

Invasive candidiasis, mainly caused by *Candida albicans*, is a serious healthcare problem with high mortality rates, particularly in immunocompromised patients. Innate immune cells express pathogen recognition receptors (PRRs) including C-type lectin-like receptors (CLRs) that bind *C*. *albicans* to initiate an immune response. Multiple CLRs including Dectin-1, Dectin-2 and Mincle have been proposed individually to contribute to the immune response to *C*. *albicans*. However how these receptors collaborate to clear a fungal infection is unknown. Herein, we used novel multi-CLR knockout (KO) mice to decipher the individual, collaborative and collective roles of Dectin-1, Dectin-2 and Mincle during systemic *C*. *albicans* infection. These studies revealed an unappreciated and profound role for CLR co-operation in anti-fungal immunity. The protective effect of multiple CLRs was markedly greater than any single receptor, and was mediated through inflammatory monocytes via recognition and phagocytosis of *C*. *albicans*, and production of *C*. *albicans*-induced cytokines and chemokines. These CLRs were dispensable for mediating similar responses from neutrophils, likely due to lower expression of these CLRs on neutrophils compared to inflammatory monocytes. Concurrent deletion of Dectin-1 and Dectin-2, or all three CLRs, resulted in dramatically increased susceptibility to systemic *C*. *albicans* infection compared to mice lacking a single CLR. Multi-CLR KO mice were unable to control fungal growth due to an inadequate early inflammatory monocyte-mediated response. In response to excessive fungal growth, the multi-CLR KO mice mounted a hyper-inflammatory response, likely leading to multiple organ failure. Thus, these data reveal a critical role for CLR co-operation in the effective control of C. *albicans* and maintenance of organ function during infection.

## Author summary

Fungal infections including invasive candidiasis are a serious healthcare problem particularly for immunocompromised patients. Mortality rates for invasive candidiasis are very high and complex anti-fungal immune responses are poorly understood, hindering the development of novel immunotherapies. Dectin-1, Dectin-2 and Mincle are three cell surface receptors that are proposed to be involved in the immune response to fungal pathogens. However, if or how these receptors work together during infection is currently unknown. Here we demonstrate that these receptors, in particular Dectin-1 and Dectin-2, work together to promote fungal clearance by a group of innate immune cells called inflammatory monocytes. Furthermore, we found that mice lacking these three receptors are dramatically susceptible to systemic *Candida albicans* infection due to defective early innate immune responses. These mice develop hyper-inflammation to try to control excessive fungal growth likely resulting in multi-organ failure. Our work helps explain how these receptors work together to clear/control invasive candidiasis. Our improved knowledge of the interactions between these receptors could be used to help design novel anti-fungal immunotherapies.

## Introduction

Fungal infections including invasive candidiasis are a serious healthcare problem particularly for patients who are immunocompromised, have undergone invasive clinical procedures or have suffered from a major trauma. Invasive candidiasis has unacceptably high mortality rates of 46–75% [1]. *Candida albicans* is the main cause of hospital acquired bloodstream *Candida* infections [2]. These infections frequently arise from gastrointestinal colonisation and migration through the mucosal barrier or from colonisation of an intravenous catheter. The early inflammatory response to systemic *Candida* infections is dominated by innate immune cells such as inflammatory monocytes, neutrophils, macrophages and dendritic cells (DCs) [3]. These cells express pathogen recognition receptors (PRRs) including C-type lectin-like receptors (CLRs), Toll-like receptors (TLRs), nucleotide-binding oligomerization domain (NOD)-like receptors (NLRs) and retinoic acid-inducible gene-I (RIG-I)-like receptors (RLRs) that bind *C*. *albicans* and initiate an immune response [4].

Multiple CLRs such as Dectin-1, Dectin-2 and Mincle have individually been proposed to participate in the immune response to *C*. *albicans*. All three receptors bind carbohydrates in the fungal cell wall, with Dectin-1 binding β-glucans and Dectin-2 and Mincle binding mannosylated ligands. All three CLRs signal through a Syk-PKCδ-Card9-NFκB pathway and through Syk-MAPK pathways [5–7]. In addition, Dectin-1 can activate NFAT, IRF1/5 and a non-canonical NFκB pathway via Raf-1 and Syk [8–10].

Dectin-1 induces multiple anti-fungal responses such as phagocytosis, cytokine production, cell recruitment, reactive oxygen species (ROS) production, canonical and non-canonical inflammasome activation and Th1 and Th17 responses [6, 9, 11–14]. In addition, Dectin-1 can prevent damaging neutrophil extracellular trap (NET) release by enhancing the phagocytosis of small yeasts, thereby minimizing pathology [15]. Dectin-1 KO mice exhibit decreased survival and increased fungal burdens during systemic *C*. *albicans* infection [14]. This protective anti-fungal effect is strain specific due to *in vivo* adaptation of the fungal cell wall [16, 17]. The importance of Dectin-1 for controlling fungal infections was confirmed by the discovery of a premature stop codon polymorphism in human Dectin-1 (*CLEC7A*), which was identified in a family with a high prevalence of mucocutaneous candidiasis and onychomycosis. This polymorphism is also associated with increased *Candida* colonisation in stem cell transplant patients and increased susceptibility to invasive aspergillosis [18–20].

Dectin-2 plays a role in phagocytosis, cytokine production, cell recruitment and Th1 and Th17 responses during systemic infection with *C*. *albicans* [6, 7, 21]. Dectin-2 KO mice display decreased survival and increased fungal burdens during systemic infection with *C*. *albicans* [7]. In addition, various polymorphisms in human Dectin-2 (*CLEC6A*), either alone or in combination with additional genes, are associated with aspergillosis and pulmonary cryptococcosis [22–24].

The function of Mincle during *C*. *albicans* infection is currently under debate. Wells *et al* showed that *C*. *albicans* induced Mincle expression, recruited Mincle to the phagocytic cup, promoted TNF production in a Mincle-dependent manner and Mincle KO mice displayed increased fungal burdens during systemic *C*. *albicans* infection [25]. However, another study showed that Mincle did not bind *C*. *albicans* or any other *Candida* spp. [26].

The Card9 pathway downstream of these three CLRs is important for *C*. *albicans*-induced cytokine production, but not zymosan internalisation [27]. In addition, *C*. *albicans*-induced Th1 and Th17 responses were defective in splenocytes from Card9 KO mice [28]. Card9 KO mice display dramatically decreased survival and increased fungal burden compared to WT controls during systemic infection with *C*. *albicans* [27]. Further, an early stop polymorphism in human *CARD9* was identified in a family where several family members suffered from recurrent fungal infections, and at least 2 family members had previously died from invasive *Candida* infection of the brain. These patients had reduced circulating IL-17^+^ T cells [29]. In addition, in a CARD9 deficient patient suffering from a chronic invasive *Candida* infection of the brain, there was a lack of *Candida*-induced cytokines from monocytes, a defect in neutrophil *Candida* killing that was independent of respiratory burst generation and reduced CD4^+^ Th17 lymphocytes [30]. Finally, Card9 has been shown to mediate neutrophil recruitment to the brain and subsequent fungal clearance during systemic infection with *C*. *albicans* in mice and in a CARD9 deficient patient [31]. The dramatic effects in Card9 KO mice and CARD9 deficient patients are likely due to the loss of signalling in response to multiple receptors including various CLRs, NLRs and TLRs.

In consideration of these findings, it is likely that the three CLRs Dectin-1, Dectin-2 and Mincle both collaborate to maintain effective CARD9 signalling and have collective independent signalling roles to control the innate immune response to *C*. *albicans* infection. Here we decipher the individual, collaborative and collective roles of Dectin-1, Dectin-2 and Mincle during systemic infection with *C*. *albicans*. Using novel multi-CLR KO mice, we found that all three receptors collaborate to bind *C*. *albicans*. Dectin-1 and Dectin-2 collaborate to induce the protective effect of inflammatory monocytes during systemic *C*. *albicans* infection while they are dispensable for various neutrophil-mediated anti-fungal responses. This is likely due to the different expression pattern of these CLRs on inflammatory monocytes and neutrophils. In the absence of Dectin-1 and Dectin-2 together or all three CLRs, these multi-CLR KO mice are unable to control fungal growth due to an inadequate early innate response. These mice then mount an excessive inflammatory response likely resulting in kidney immunopathology and multi-organ failure. Together these CLRs mediate the various immune responses required for clearance of C. *albicans* and to maintain organ function during infection.

## Materials and methods

### Mice

CLR KO mice used in this study are shown in Table 1. Dectin-1 KO mice [14] generated on a 129 background were backcrossed for a total of 11 generations to the C57BL6/J background. Mincle KO mice [25] were obtained from the Mutant Mouse Resource and Research Center (MMRRC). Dectin-2 KO mice [7] were sourced from Tokyo University of Science. As the Dectin-2 gene cluster (*Clec4n*, *Clec4d* and *Clec4e*) and Dectin-1 (*Clec7a*) are located on mouse chromosome 6, ~6cM apart (S1 Fig), a unique targeting strategy involving cross-breeding to identify rare recombination events and Crispr-Cas9 technology was employed to generate double (DKO) and triple (TKO) KO mice. The various multi-CLR KO mouse strains were then bred to homozygosity. Mincle-Dectin-1 DKO1 mice were generated by crossing Mincle KO with Dectin-1 KO mice. Sigma Advanced Genetic Engineering (SAGE) generated Mincle-Dectin-2 DKO2 mice by targeting Dectin-2 in Mincle KO mice using Crispr-Cas9 technology. Briefly, SAGE designed donor plasmids with homologous arms and an insert sequence containing 3-frame stop codons upstream of a LoxP site. The following sequence (GAGTGACTGATAACTTCGTATAGCATACATTATACGAAGTTATCGAT) was inserted into exon 3 of *Clec4n* (Dectin-2) in Mincle KO mice (S1 Fig). Dectin-1-Dectin-2 DKO mice were generated by crossing Dectin-1 KO with Dectin-2 KO mice. Mincle-Dectin-2-Dectin-1 TKO1 mice were generated by crossing Mincle-Dectin-2 DKO2 mice with Dectin-1 mice (Table 1). Mice were age and gender matched for experiments. Female mice were co-housed for 1–3 weeks prior to *in vivo* experiments. For various experiments where male mice were used, bedding was swapped between cages for 3–7 days prior to experiments.

**Table 1:** CLR KO mice used in this study.

Mice Strain	Method of Generation/Source
**Dectin-1 (D1) KO**	Taylor *et al* [14]- Backcrossed 11 generations to C57BL6/J background
**Mincle (Min) KO**	Wells *et al* [25]—Imported from MMRRC (USA)
**Dectin-2 (D2) KO**	Saijo *et al* [7]- Imported from Yoichiro Iwakura (Tokyo University of Science)
**Mincle-Dectin-1 DKO1 (Min-D1 DKO1)**	Crossed Mincle KO with Dectin-1 KO -rare recombination event ~**1/50 mice**
**Mincle-Dectin-2 DKO2 (Min-D2 DKO2)**	Inserted LoxP site and 3 stop codons using Crispr-Cas9 technology into *Clec4n* (Dectin-2) in Mincle KO mice.
**Dectin-1-Dectin-2 DKO (D1-D2 DKO)**	Crossed Dectin-2 KO with Dectin-1 KO–rare recombination event ~**1/50 mice**.
**Mincle-Dectin-2-Dectin-1 TKO1 (Min-D2-D1 TKO1)**	Crossed Mincle-Dectin-2 DKO2 with Dectin-1 KO–rare recombination event **(**~**1/50 mice)**

### Ethics

The mice were maintained and handled according to institutional and UK Home Office guidelines. This study was performed in strict accordance with the Project License (30/2938, P05D6A456) and procedures that were approved by Cardiff University Animal Welfare and Ethical Review Body and the UK Home Office. The animal care and use protocol adhered to the Animals (Scientific Procedures) Act 1986.

### Reagents

Flow cytometry antibodies against Ly6C, Ly6G, CD11b, F4/80, CD11c, MHCII, B220, CD19, CD3, CD4, CD8, CD49b, TLR2, TLR4, mannose receptor (MR), IFN-γ, IL-17, TNF and isotype controls were purchased from Biolegend. Dectin-1 antibody (AbD Serotec), Dectin-2 antibody D2.11E4 [32] and Mincle/Clec4e antibody (Abnova) were used in this study. IFN-γ, IL-17, G-CSF, MIP2, MIP1α and MCP1 ELISAs were purchased from R&D while IL-1β, IL-6, IL-10, IL-12p40 and TNF ELISAs were purchased from Invitrogen.

### *Candida* culture

*Candida albicans* SC5314 (ATCC) was plated on YPD agar, cultured for 16–20 h in YPD broth, washed three times with PBS and resuspended at the required concentration in PBS.

### *In vivo* Systemic *Candida* infections

Age matched (8–12 weeks old) female mice were injected *i*.*v*. with 100μl of *C*. *albicans* in PBS. Mice were monitored and weighed daily. Experiments were continued for a maximum of 30 days post-infection. Kidneys, brains and spleens were harvested. The left kidney was placed in PBS, homogenised and serial dilutions were plated on YPD agar containing 50μg/ml chloramphenicol. The plates were cultured for 24 h and CFU were calculated per g organ. Alternatively, the left kidney was diced into small pieces, incubated at 37°C for 30 min in DMEM/F12 medium (Gibco) including 0.2mg/ml Liberase TL (Roche) and 100U/ml DNase I (Invitrogen). Following digestion, DMEM/F12 medium containing 10% fetal bovine serum (FBS) was added, filtered through 40μm cell strainers (BD) and then centrifuged at 4°C, 500g for 5 min. The pellet was resuspended with ice-cold PBS as a single cell suspension for further staining procedures. The right kidney was placed in 10% formalin, embedded in paraffin wax blocks, processed using an automated tissue processor, sectioned at 4μm, deparaffinised and stained for H&E and PAS according to standard protocols. The spleens were homogenized, red blood cells were lysed with ACK lysis buffer and the cells were washed with PBS and IMDM. Cells were resuspended in IMDM containing 10% FBS, 2mM L-glutamine, penicillin/streptomycin, 50μM 2-mercaptoethanol plated and restimulated with *C*. *albicans* for 48 h in the presence of Amphotericin B or with PMA (50ng/ml) and Ionomycin (0.5μg/ml) in the presence of 0.2% Brefeldin A for 4 h. IFN-γ and IL-17 levels were measured by ELISA in *C*. *albicans*-stimulated cells while PMA/Ionomycin-induced IFN-γ and IL-17A producing cells were analysed by flow cytometry. Alternatively, the splenocytes were fixed in 1% paraformaldehyde for 20 min after the red blood lysis and the PBS wash. The cells were centrifuged and resuspended in PBS. The cells were then stained with antibodies for myeloid cell markers and analysed by flow cytometry.

### Histology Scoring

Kidneys were examined at 4μm and the cortex and medulla individually assessed for the presence of inflammatory cells including neutrophils and lymphocytes / plasma cells and scored as follows: Score 0 = no inflammation, Score 1 = < 3 foci of inflammation, Score 2 = 4 to 6 foci of inflammation, Score 3 = > 6 foci of inflammation, but less than 25% of kidney affected, Score 4 = > 25% of kidney affected. Scoring was performed by a pathologist blinded to the experimental groups.

### Primary myeloid cells

To recruit primary myeloid cells, mice were injected intraperitoneally with 0.5ml 2% (w/v) BIOgel P-100 polyacrylamide beads (BIO-Rad), 16–18 h prior to peritoneal lavage. Inflammatory cells were collected after sacrifice by peritoneal lavage with ice-cold RPMI containing 10% FBS and 1% penicillin/streptomycin. To remove BIOgel beads from cells, the cells were filtered through 40μm cell strainers. Cells were washed twice with RPMI containing 10% FBS and 1% penicillin/streptomycin.

### Fluorescent labelling of *C*. *albicans*

*C*. *albicans* was washed three times with PBS and resuspended at 1x10^8^/ml in PBS. *Candida* was labelled with cell trace far red (Invitrogen) at a concentration of 3.4μg/ml for 30 min while rotating at RT. *Candida* was washed three times with PBS and resuspended at the desired concentration in RPMI containing 10% FBS and 1% penicillin/streptomycin.

### *C*. *albicans* recognition and dihydrorhodamine 123 ROS assay

4x10^5^ BIOgel recruited inflammatory cells were plated in 100μl RPMI containing 10% FBS and 1% penicillin/streptomycin in ultra-low attachment 96 well round bottom plates. 4x10^5^ cell trace far red labelled *C*. *albicans* in 100μl RPMI containing 10% FBS and 1% penicillin/streptomycin were added to the inflammatory cells and incubated at 37°C for 15 min. Cells were centrifuged and supernatants were removed. 100μl of 5μM dihydrorhodamine 123 (DHR-123) (Invitrogen) in warmed PBS was added to cells and incubated at 37°C for 15 min. Cells were centrifuged, supernatants were removed, and cells were washed once with ice-cold PBS. Cells were stained with antibodies for Ly6G and CD11b and analysed by flow cytometry or Amnis Imagestream^*x*^ MkII.

### Luminol ROS assay and candidacidal assay

BIOgel recruited inflammatory cells were recovered by peritoneal lavage and washed as described above. Cells were stained with antibodies against Ly6G, CD19 and CD11b. Ly6G^-^CD11b^+^CD19^-^ inflammatory monocytes/macrophages and Ly6G^+^CD11b^+^CD19^-^ neutrophils were purified by cell sorting on a FACS Aria cell sorter.

To opsonise *C*. *albicans*, 5x10^7^ *C*. *albicans* were placed in a 1.5ml Eppendorf tube and resuspended in 200μl mouse serum. This was incubated at 37°C for 15 min with frequent mixing. Opsonised *C*. *albicans* was washed 3x with PBS prior to use.

For the luminol assay, purified cells were seeded at 1x10^5^ cells per well in a 96-well luminescence plate in 100μl DMEM with no phenol red containing 10% FBS, 2mM L-glutamine and 800μM luminol. The plate was incubated for 30 min at 37°C, after which 100μl media, 1x10^5^ *C*. *albicans*, opsonised *C*. *albicans* or PMA (400ng/ml) was added. The plate was immediately placed in a luminescence plate reader at 37°C and luminescence readings were measured every 8.7 min for 3.5 h.

For the candidacidal assay, purified cells were seeded at 1x10^5^ cells per well in a 96-well round bottom plate in 100μl DMEM with no phenol red containing 10% FBS and 2mM L-glutamine. 100μl media, 1x10^5^ *C*. *albicans* or opsonised *C*. *albicans* was added. The plate was incubated for 3 h at 37°C. Cells were lysed with 200μl 1% Triton X-100, and *Candida* were plated on YPD agar and incubated at 37°C for 24 h. The percentage of *Candida* killed by monocytes/macrophages or neutrophils was determined by [1-(number of *C*. *albicans* incubated with cells)/(number of *C*. *albicans* incubated without cells)]x100.

### *C*. *albicans*-induced intracellular cytokine

4x10^5^ BIOgel recruited inflammatory cells were plated in 100μl RPMI containing 10% FBS and 1% penicillin/streptomycin in ultra low attachment 96 well round bottom plates. 4x10^5^ cell trace far red labelled *C*. *albicans* in 100μl RPMI containing 10% FBS and 1% penicillin/streptomycin were added to the inflammatory cells and incubated at 37°C for 3 h in the presence of 0.2% Brefeldin A. Cells were stained with live/dead fixable aqua dead cell stain kit (Invitrogen), antibodies against Ly6G, CD11b and CD19 and intracellular TNF levels were measured by flow cytometry.

### *C*. *albicans* recognition and internalisation

BIOgel recruited inflammatory cells were stained with antibodies against Ly6G and CD11b. Cells and cell trace far red labelled *C*. *albicans* were mixed at 1:1 ratio and analysed by Amnis Imagestream^*x*^ MkII over 1.5 h at RT. An object mask on CD11b and a morphology mask on *C*. *albicans* was applied on pre-gated neutrophils or monocytes which were best in focus (Gradient RMS_M01_BRF) using Ideas software 6.2. An internalisation feature was used [Internalisation_Object(M02, CD11b, Tight)_*C*. *albicans*] to differentiate between cells that had internalised *C*. *albicans* versus cells that had *C*. *albicans* bound externally.

### RNAseq

BIOgel recruited inflammatory cells were recovered by peritoneal lavage and washed as described above. Cells were stained with antibodies against Ly6G, CD4, CD8, Ly6C, F4/80, CD19, DX5 and CD11b. Ly6C^hi^Ly6G^-^CD11b^+^F4/80^+^CD4^-^CD8^-^DX5^-^CD19^-^ inflammatory monocytes were purified by cell sorting on a FACS Aria cell sorter. Inflammatory monocytes were incubated at 37°C with *C*. *albicans* at 3:1 Cells:*Candida* ratio for 3 h in RPMI containing 10% FBS, 1% penicillin/streptomycin, 1% L-glutamine, 0.5% HEPES and 50μM 2-mercaptoethanol. Cell pellets were snap frozen and stored in -80°C. RNA was extracted using a RNeasy mini kit (Qiagen). Total RNA quality and quantity was assessed using Agilent 2100 Bioanalyser and a RNA Nano 6000 kit (Agilent Technologies). Total RNA with a RIN value >7 was depleted of ribosomal RNA using the NEBNext rRNA Depletion Kit (Human/Mouse/Rat), (New England BioLabs, NEB) and the sequencing libraries were prepared using the NEB Ultra II Directional RNA Library Prep Kit for Illumina (NEB). Libraries were normalised to 10nM, pooled and sequenced using a 75-base paired-end (2x75bp PE) dual index read format on the Illumina HiSeq4000 according to the manufacturer’s instructions. Reads from sequencing were trimmed with Trimmomatic [33] and assessed for quality using FastQC (https://www.bioinformatics.babraham.ac.uk/projects/fastqc/), using default parameters. Reads were mapped to the mouse GRCm38 reference genome using STAR [34] and counts were assigned to transcripts using featureCounts [35] using the GRCm38.84 Ensembl gene build GTF. Both the reference genome and GTF were downloaded from the Ensembl FTP site (http://www.ensembl.org/info/data/ftp/index.html/). RNAseq data has been deposited to ArrayExpress (https://www.ebi.ac.uk/arrayexpress/) under accession number E-MTAB-8030. Differential gene expression analyses used the DESeq2 Bioconductor package [36]. Genes were filtered from the analysis where read count < 10 over all replicates and conditions. Normalised gene expression values (FPKM), used for downstream cluster analysis, were calculated for all significantly differentially expressed genes (significance: adjusted p value < 0.05, Benjamini-Hochberg correction for multiple testing). Cluster analysis were performed in Genesis [37] where FPKM count data were z-score transformed over genes. Pathway term-enrichment analyses of each cluster was performed in Ingenuity IPA (QIAGEN: Inc. https://www.qiagenbioinformatics.com/products/ingenuity-pathway-analysis)

### Cytokine/Chemokine assays

Bone marrow cells were flushed out of the femurs and tibiae of mice. Red blood cells were lysed with ACK lysis buffer and the cells were washed with PBS. Monocytes were magnetically sorted using a negative selection bone marrow monocyte isolation kit. Cells were incubated with *C*. *albicans* at 3:1 Cells:*Candida* ratio for 24 h. 2.5μg/ml Amphotericin B was added 2 h after stimulation. Cell culture supernatants were recovered and assayed for cytokine/chemokine by ELISA (Invitrogen/R&D).

### *In vivo* Peritoneal *C*. *albicans* infections

Age matched male mice were injected *i*.*p*. with 1x10^5^ CFU of *C*. *albicans* in 100μl PBS, 4 h prior to peritoneal lavage. Inflammatory cells were collected after sacrifice by peritoneal lavage with 1ml ice-cold PBS. Cells were counted using a MUSE cell counter, cells were centrifuged and lavage fluid was removed and placed in a fresh 1.5ml eppendorf tube and frozen at -80°C. Cells were stained with antibodies against Ly6G, Ly6C, CD11b, F4/80, CD11c, MHCII and CD19. Myeloid cell recruitment was measured by flow cytometry. Cytokine/chemokine levels in the lavage fluid were measured by ELISA.

### Adoptive transfer

Bone marrow cells were flushed out of the femurs and tibiae of mice. Red blood cells were lysed with ACK lysis buffer and the cells were washed with PBS. Monocytes were magnetically sorted using a negative selection bone marrow monocyte isolation kit. Monocytes were further purified to Ly6C^hi^Ly6G^-^CD11b^+^CD4^-^CD8^-^CD19^-^CD49b^-^Cd11c^-^ cells by FACS sorting on a FACS Aria cell sorter. Cells were 93–99% viable post-sort. 3-4x10^6^ Ly6C^hi^ monocytes from wildtype (WT) or Min-D2-D1 TKO1 mice were injected intravenously into Ccr2 KO mice followed by i.v. injection of 1.5x10^5^ CFU of *C*. *albicans* in PBS. Mice were weighed and monitored daily for 3 days. Kidneys and brains were placed in PBS, homogenised and serial dilutions were plated on YPD agar containing 50μg/ml chloramphenicol. The plates were cultured for 24 h and CFU were calculated per g organ.

### Adoptive transfer of labelled cells

Bone marrow cells were flushed out of the femurs and tibiae of mice. Red blood cells were lysed with ACK lysis buffer and the cells were washed with PBS. Monocytes were magnetically sorted using a negative selection bone marrow monocyte isolation kit. Cells were labelled with 5μM CFSE in PBS containing 0.1% BSA for 10 min at 37°C. RPMI containing 10% FBS was added to the cells and cells were incubated on ice for 5 min. Cells were washed with PBS and 2.5x10^6^ monocytes from WT or Min-D2-D1 TKO1 mice were injected intravenously into Ccr2 KO mice followed by i.v. injection of 1.5x10^5^ CFU of *C*. *albicans* in PBS. After 24 h, kidneys were placed in PBS. The left kidney was diced into small pieces, incubated at 37°C for 30 min in DMEM/F12 medium including 0.2mg/ml Liberase TL and 100U/ml DNase I. Following digestion, DMEM/F12 medium containing 10% FBS was added, filtered through 40μm cell strainers and then centrifuged at 4°C, 500g for 5 min. The pellet was resuspended with ice-cold PBS as a single cell suspension for further staining procedures. The right kidney was homogenised and serial dilutions were plated on YPD agar containing 50μg/ml chloramphenicol. The plates were cultured for 24 h and CFU were calculated per g organ. Chemokine levels in the kidney homogenates were determined by ELISA.

### Statistical Methods

Data are presented as means +/- s.e.m. and are representative or cumulative data from the indicated number of independent experiments. Data was tested for normality and if data followed a Gaussian distribution then one-way ANOVA followed by Bonferroni’s post-test or 2-way ANOVA was used for statistical analysis when multiple groups were analysed or Student’s *t* test was used for statistical analysis when two groups were analysed. A Gaussian distribution was assumed for experiments with small sample numbers. When data did not follow a Gaussian distribution, it was transformed by Y = sqrt(Y+0.5) for data containing zeros or by Y = logY for other data [38] and analysed by Student’s *t* test or one-way ANOVA followed by Bonferroni’s post-test if it then followed a Gaussian distribution. If data still did not follow a Gaussian distribution it was analysed by non-parametric Mann-Whitney test or Kruskal-Wallis test followed by Dunn’s post-test. Statistical significance was set at *p<0.05 **p<0.005 ***p<0.001.

## Results

### Multi-CLR KO mice are dramatically susceptible to *C*. *albicans* infection

As Dectin-1, Dectin-2 and Mincle have individually been proposed to play roles in response to *C*. *albicans* [7, 14, 25], we aimed to identify collaborative and redundant roles of these CLRs. To this end we generated mice deficient in different combinations of these CLRs (Table 1). We confirmed appropriate loss of CLR protein expression in bone marrow derived dendritic cells (BMDCs) (S1 Fig), inflammatory monocytes/macrophages (S1 Fig) and neutrophils (S1 Fig) from the multi-CLR KO mice generated in this study. Inflammatory monocytes/macrophages displayed higher expression of Dectin-1 and Dectin-2 than neutrophils while Mincle expression was higher on neutrophils (S1 Fig). As expected, expression of additional receptors such as MR, TLR2, TLR4 and CD14 was unaffected on inflammatory monocytes/macrophages from the various multi-CLR KO mice (S1 Fig). The multi-CLR KO mice were viable, had no gross abnormalities and had normal differential splenocyte counts (S1 Table).

**Fig 1:**
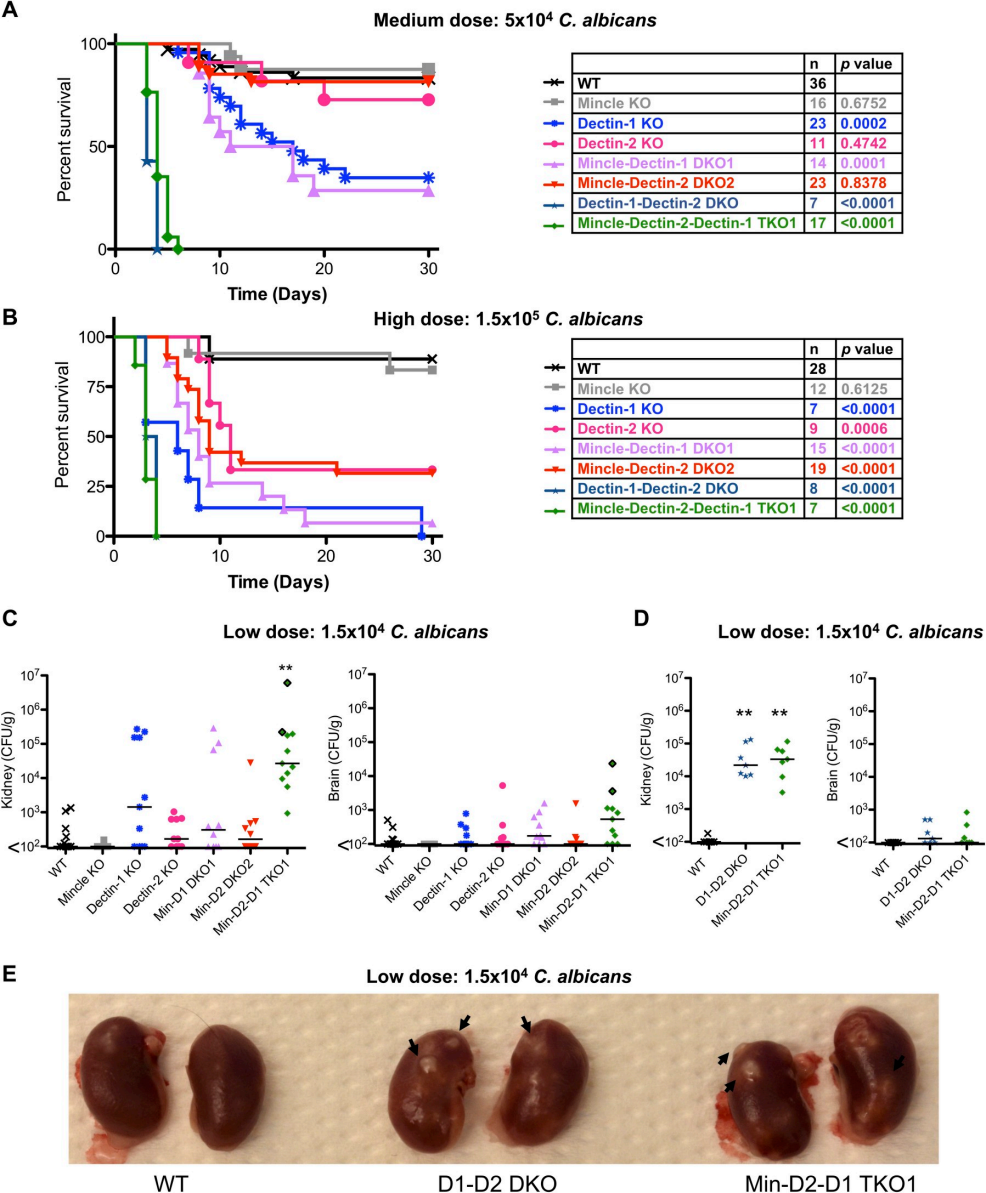
Multi-CLR KO mice are dramatically susceptible to *C*. *albicans* infection. (A-B) Survival curves based on humane end point of WT and multi-CLR KO mice infected intravenously with 5x10^4^ CFU (A) or 1.5x10^5^ CFU (B) *C*. *albicans* SC5314. Graphs show cumulative data from 7 (A) or 6 (B) independent experiments. The total number of mice and *p* values for survival curves of CLR KO mice compared to WT mice by log-rank test are shown in the tables opposite each graph. (C-D) Quantification of fungal burden in the kidneys (left) or brains (right) of WT and multi-CLR KO mice 7 days (C) or 6 days (D) after intravenous infection with 1.5x10^4^ CFU *C*. *albicans*. Graphs display cumulative data from 3 (C) or 2 (D) independent experiments. Each symbol represents one mouse. Two Mincle-Dectin-2-Dectin-1 TKO1 mice had to be taken on day 6 post-infection (C). The symbols for these two mice have a black outline. (Kruskal-Wallis test with Dunn’s post-test on transformed data). (E) Photograph of representative kidneys from WT and multi-CLR KO mice 6 days after intravenous infection with 1.5x10^4^ CFU *C*. *albicans*.

In order to determine the collective and contributing roles of these three CLRs during systemic infection with *C*. *albicans*, we injected groups of WT and multi-CLR KO mice intravenously with low (1.5x10^4^ CFU), medium (5x10^4^ CFU) or high (1.5x10^5^ CFU) doses of *C*. *albicans* SC5314. Dectin-1-Dectin-2 DKO and Mincle-Dectin-2-Dectin-1 TKO1 mice displayed marked susceptibility to systemic infection with *C*. *albicans* with both medium (Fig 1A) and high (Fig 1B) dose infection. All Dectin-1-Dectin-2 DKO and Mincle-Dectin-2-Dectin-1 TKO1 mice infected with a medium or high dose of *C*. *albicans* succumbed to the infection by day 6 (by humane end-point) and some Dectin-1-Dectin-2 DKO and Mincle-Dectin-2-Dectin-1 TKO1 mice infected with a low dose of *C*. *albicans* even succumbed within 6 days of infection (Fig 1C). Dectin-1 and Mincle-Dectin-1 DKO1 mice showed significant susceptibility to systemic infection with *C*. *albicans* with both medium (Fig 1A) and high (Fig 1B) dose, however this effect was much less profound than that observed in Dectin-1-Dectin-2 DKO and Mincle-Dectin-2-Dectin-1 TKO1 mice. Dectin-2 and Mincle-Dectin-2 DKO2 mice only showed increased susceptibility to systemic infection with *C*. *albicans* with high dose infection (Fig 1B) but not with medium dose infection (Fig 1A). In comparison, Mincle KO mice did not show substantial susceptibility to systemic infection with *C*. *albicans* with any of the tested doses (Fig 1A and 1B). The CLR KO mice with highest susceptibility (Dectin-1-Dectin-2 DKO and Mincle-Dectin-2-Dectin-1 TKO1) to systemic infection with *C*. *albicans* (Fig 1A and 1B) displayed the highest fungal burden in the kidneys and brains with low dose infection 6–7 days post-infection (Fig 1C and 1D). Similar effects were observed in mice infected with medium dose (S2 Fig) or high dose (S2 Fig) infection at humane end point or 30 days post-infection. Nodules of *Candida* were visibly evident on the kidneys of Dectin-1-Dectin-2 DKO and Mincle-Dectin-2-Dectin-1 TKO1 mice (Fig 1E). These data indicate that of the single KO mice, Dectin-1 is most important for *C*. *albicans* clearance followed by Dectin-2, and loss of both of these receptors severely diminishes control of systemic *C*. *albicans* infection. Mincle deficiency alone or in combination with Dectin-1, Dectin-2 or Dectin-1 and Dectin-2 had limited impact indicating that Mincle is dispensable for clearance of systemic infection.

### *C*. *albicans*-infected multi-CLR KO mice display enhanced inflammation in association with uncontrolled fungal burden

**Fig 2:**
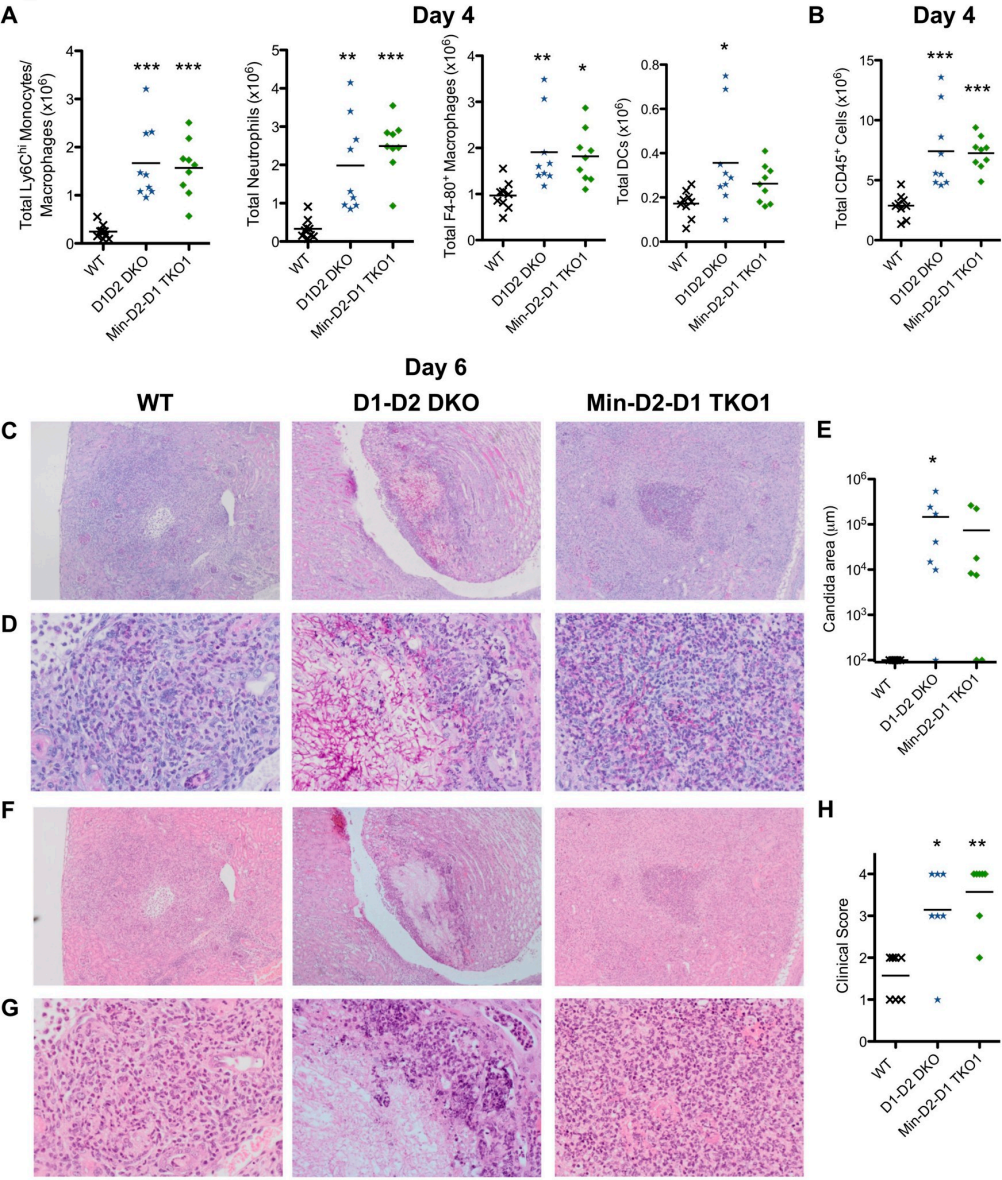
*C*. *albicans*-infected multi-CLR KO mice display enhanced inflammation in association with uncontrolled fungal burden. (A) Total myeloid cell numbers in the CD45^+^ cells and (B) total CD45^+^ cells from kidneys of WT and multi-CLR KO mice 4 days post-infection with 1.5x10^4^ CFU *C*. *albicans* were quantified by flow cytometry. Graphs display the cumulative data from 2 independent experiments. Each symbol represents an individual mouse. (1-way ANOVA with Bonferroni’s post-test, Total neutrophils: Kruskal-Wallis test with Dunn’s post-test). (C-D, E-F) Representative WT (left panels), D1-D2 DKO (middle panels) and Min-D2-D1 TKO1 (right panels) kidneys, 6 days after *i*.*v*. infection with 1.5x10^4^ CFU *C*. *albicans*. (C-D) Kidney sections were stained with Periodic acid Schiff reagent or (E-F) hematoxylin and eosin. Images shown are 4x magnification (C & F) and 20x magnification (D & G). Graphs display *Candida* area (μm) (E) and clinical score (H). Graphs are the cumulative result of 2 independent experiments. Each symbol represents an individual mouse. (1-way ANOVA with Bonferroni’s post-test: Performed on transformed data for *Candida* area).

As the Dectin-1-Dectin-2 DKO and Mincle-Dectin-2-Dectin-1 TKO1 mice displayed increased susceptibility to systemic *C*. *albicans* infection and increased fungal burden, we next examined immune cell recruitment to the kidneys of these mice. We observed increased numbers of myeloid cells including Ly6C^hi^ monocytes/macrophages and neutrophils in the kidneys of Dectin-1-Dectin-2 DKO and Mincle-Dectin-2-Dectin-1 TKO1 mice compared to WT mice as early as 20 h post-infection (S3 Fig) and also at 4 days post-infection (Fig 2A). In agreement with this, the total number of CD45^+^ cells was increased in the kidneys of the multi-CLR KO mice compared to WT mice (Fig 2B). By day 6 post-infection, we observed that WT mice almost completely cleared low dose *C*. *albicans* from their kidneys while Dectin-1-Dectin-2 DKO and Mincle-Dectin-2-Dectin-1 TKO1 mice displayed large areas/abscesses of *Candida* growth, sometimes in hyphal form (Fig 2C–2E). While kidneys from WT mice displayed limited and predominantly dispersed chronic inflammation with sparse neutrophils 6 days post-infection, kidneys from Dectin-1-Dectin-2 DKO and Mincle-Dectin-2-Dectin-1 TKO1 mice displayed extensive mixed inflammation affecting the cortex, medulla and renal pelvis with focal extension into surrounding adipose tissue (Fig 2F–2H). These multi-CLR KO kidneys showed multi-focal suppurative granulomatous inflammation containing central neutrophilic microabscesses with frequent *Candida* identification, surrounded by macrophages and chronic inflammatory cells. In some multi-CLR KO cases, occasional kidneys developed cystic degeneration.

Similarly, at 4 days post-infection, we observed increased numbers of splenic myeloid cell populations including Ly6C^hi^ monocytes and neutrophils and total splenocytes in the susceptible mice strains compared to WT mice (S4 Fig). By 6 days post-infection, splenocyte numbers in the multi-CLR KO mice continued to increase compared to WT mice (S4 Fig). This was not observed in the other single or double KO strains (S4 Fig). At 6 days post-infection the total number of T cells, CD8^+^ T cells and/or NK cells were also increased in these multi-CLR KO mice (S4 Fig). We then restimulated splenocytes from these infected mice with PMA and Ionomycin, to examine the ability of these cells to produce IFN-γ and IL-17. This mainly induced an IFN-γ response with a much smaller IL-17 response. Due to the splenomegaly in Dectin-1-Dectin-2 DKO and Mincle-Dectin-2-Dectin-1 TKO1 mice, the total number of IFN-γ producing cells were increased (S4 Fig). Upon restimulation of the splenocytes with live *C*. *albicans*, the multi-CLR KO mice displayed a trend towards increased IFN-γ production although this did not always reach significance (S4 Fig).

These kidney and splenic data indicate that uncontrolled fungal growth in Dectin-1-Dectin-2 DKO and Mincle-Dectin-2-Dectin-1 TKO1 mice is associated with persistent/prolonged inflammatory cell recruitment which may contribute to tissue immunopathology and organ failure.

### CLR collaboration mediates *C*. *albicans* recognition by inflammatory monocytes/macrophages but not neutrophils

**Fig 3:**
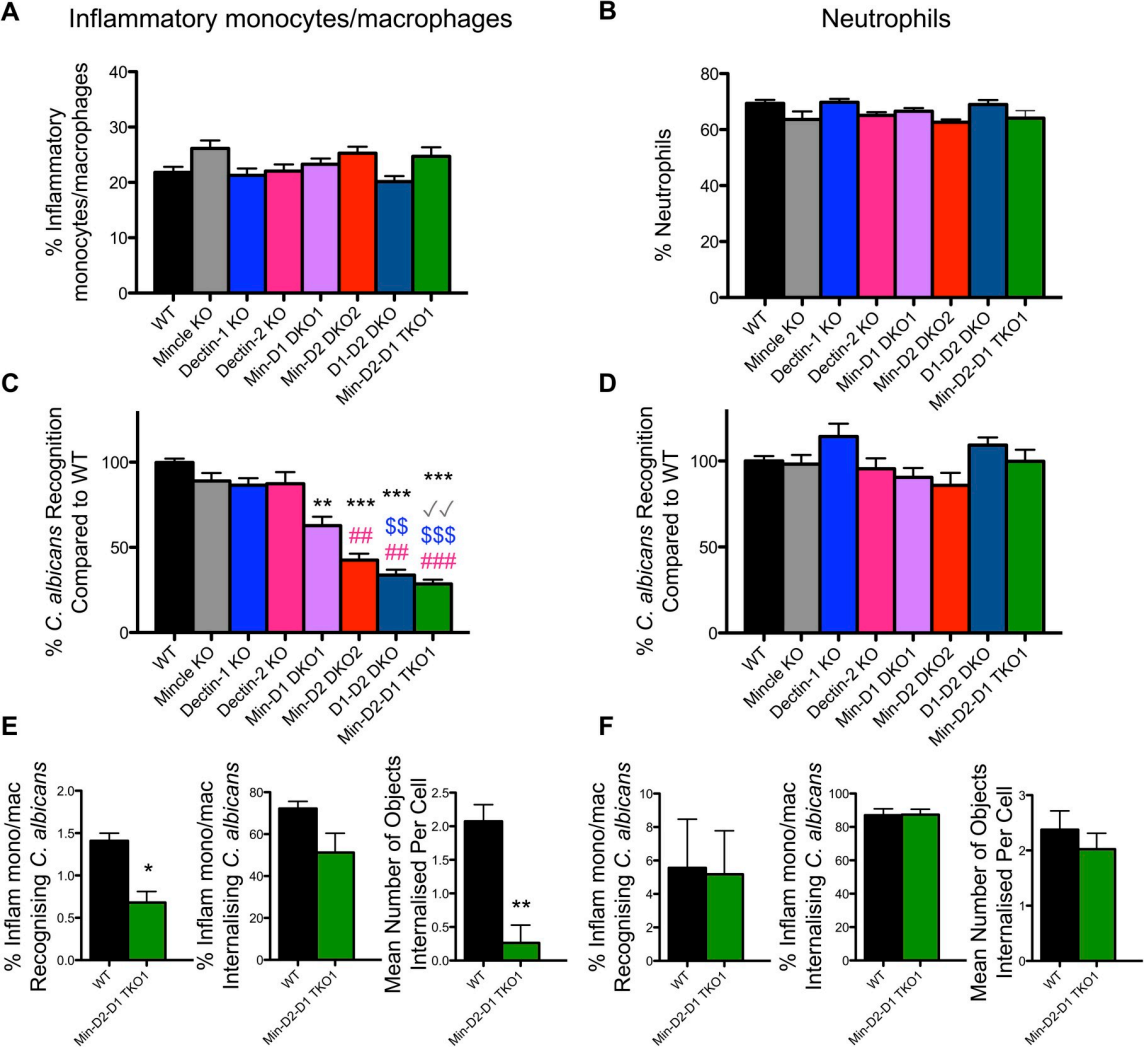
CLR collaboration mediates *C*. *albicans* recognition by inflammatory monocytes/macrophages but not neutrophils. (A-F) WT and CLR KO mice were injected with BIOgel i.p. and inflammatory cells were recovered by peritoneal lavage after 16–18 h. (A-D) Cells were stimulated for 15 min at 37°C with cell trace far red-labelled *C*. *albicans* at a 1:1 ratio. Cells were stained with anti-Ly6G and anti-CD11b. (A-B) Graphs display % recruited Ly6G^-^CD11b^+^ inflammatory monocytes/macrophages (A) and % Ly6G^+^CD11b^+^ neutrophils (B) in the peritoneal cavity 16–18 h after BIOgel injection. (1-way ANOVA with Bonferroni’s post-test: Performed on transformed data for Inflammatory monocytes/macrophages) (C) Ly6G^-^CD11b^+^ inflammatory monocytes/macrophages and (D) Ly6G^+^CD11b^+^ neutrophils that interacted with *C*. *albicans* were measured by flow cytometry. Percentage WT cells that recognised *C*. *albicans* was set at 100%. n = 7–16 mice. Graphs show cumulative data from 8 independent experiments. (Inflammatory monocytes/macrophages: Kruskal-Wallis test with Dunn’s post-test; Neutrophils: 1-way ANOVA with Bonferroni’s post-test). * compared to WT, √ compared to Mincle KO, $ compared to Dectin-1 KO, # compared to Dectin-2 KO (E-F) WT and Min-D2-D1 TKO1 cells were stained with anti-Ly6G and anti-CD11b. Cells were stimulated with cell trace far red-labelled *C*. *albicans* at a 1:1 ratio for 1.5 h and analysed by image flow cytometry (Amnis Imagestream^*x*^ MkII) for the course of the 1.5 h stimulation at room temperature. Inflammatory monocytes/macrophages (E) and neutrophils (F) that interacted with labelled *C*. *albicans*, internalised *C*. *albicans* and the mean number of objects internalised per cell that recognised *C*. *albicans* were quantified. n = 3 mice. (Student’s paired *t* test: % Inflam mono/mac recognising *C*. *albicans* on normalised data). (E-F) Graphs show the cumulative results from 3 independent experiments.

As inflammatory monocytes, macrophages and neutrophils contribute to protection during the early stages of systemic infection with *C*. *albicans* [39, 40], we examined the collective and contributing roles of Dectin-1, Dectin-2 and Mincle to *C*. *albicans* recognition by myeloid cells. BIOgel polyacrylamide beads were injected intraperitoneally to elucidate inflammatory myeloid cells including neutrophils and monocytes/macrophages [41]. The different KO strains demonstrated similar recruitment of inflammatory monocytes/macrophages (Fig 3A) and neutrophils (Fig 3B) in response to BIOgel. These cells were recovered from the peritoneal cavity of WT and multi-CLR KO mice and incubated *ex vivo* with cell trace far red-labelled live *C*. *albicans*. The percentage of inflammatory monocytes/macrophages (Ly6G^-^CD11b^+^) and neutrophils (Ly6G^+^CD11b^+^) interacting with *C*. *albicans* were measured by flow cytometry. The inflammatory monocyte/macrophage cell population is Ly6G^-^CD11b^+^Ly6C^hi/lo^F4-80^+^ (S5 Fig). Inflammatory monocytes/macrophages lacking Dectin-1, Dectin-2 or Mincle individually showed no major defects in *C*. *albicans* recognition after 15 min incubation. However, inflammatory monocytes/macrophages lacking any two or all three of these CLRs showed significant defects in *C*. *albicans* recognition/binding compared to WT cells (Fig 3C and S5 Fig). In addition, Mincle-Dectin-2 DKO2 cells, Dectin-1-Dectin-2 DKO and Mincle-Dectin-2-Dectin-1 TKO1 inflammatory monocytes/macrophages showed significant defects in *C*. *albicans* binding compared to Dectin-2 KO cells, while Dectin-1-Dectin-2 DKO and Mincle-Dectin-2-Dectin-1 TKO1 inflammatory monocytes/macrophages showed significant defects in *C*. *albicans* binding compared to Dectin-1 KO cells (Fig 3C). In contrast, neutrophils deficient in expression of individual or multiple CLRs showed no major defects in *C*. *albicans* binding (Fig 3D and S5 Fig) suggesting the involvement of additional receptors. Using an Amnis Imagestream^*x*^ MkII, we confirmed a similar trend towards reduced recognition and internalisation of *C*. *albicans* by Mincle-Dectin-2-Dectin-1 TKO1 inflammatory monocytes/macrophages over 1.5 h (Fig 3E and S6 Fig). In addition, we found that the mean number of *C*. *albicans* yeast particles internalised per WT inflammatory monocyte/macrophage is ~4 times greater than the mean number of *C*. *albicans* yeast particles internalised per Mincle-Dectin-2-Dectin-1 TKO1 inflammatory monocyte/macrophage (Fig 3E). However, no major differences were found for recognition or internalisation by neutrophils (Fig 3F and S6 Fig). Some of the differences between inflammatory monocytes/macrophages and neutrophils are likely due to differences in expression of the CLRs (S1 Fig) and other receptors between these cell types. These data indicate that while Mincle is not required for clearance of *C*. *albicans in vivo*, it still contributes to recognition of *C*. *albicans* by inflammatory monocytes/macrophages in addition to the main CLRs, Dectin-1 and Dectin-2, while additional receptors are likely to be important for this function in neutrophils.

### Monocytes/macrophages and neutrophils display limited dependence on CLRs for ROS production

**Fig 4:**
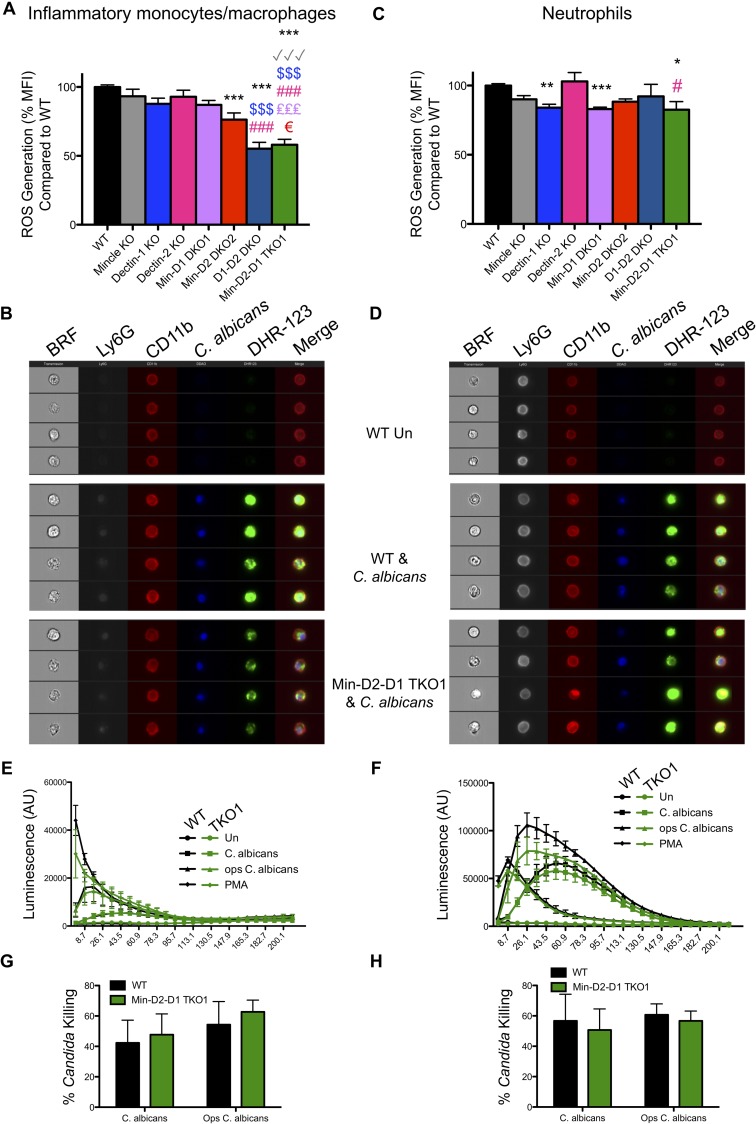
Monocytes/macrophages and neutrophils display limited dependence on CLRs for ROS production. (A-D) WT and CLR KO mice were injected with BIOgel i.p. and inflammatory cells were recovered by peritoneal lavage after 16–18 h. Cells were stimulated for 15 min with cell trace far red-labelled *C*. *albicans* at a 1:1 ratio. Cells were loaded with the ROS indicator Dihydrorhodamine 123 (DHR-123) and stained with anti-Ly6G and anti-CD11b. (A & C) ROS production (DHR-123) was measured by flow cytometry in inflammatory monocytes/macrophages (A) and neutrophils (C) that interacted with labelled *C*. *albicans*. DHR-123 MFI for WT cells interacting with *C*. *albicans* was set at 100%. n = 7–16 mice. Graphs show cumulative data from 8 independent experiments. (Inflammatory monocytes/macrophages: 1-way ANOVA with Bonferroni’s post-test; Neutrophils: Kruskal-Wallis test with Dunn’s post-test). * compared to WT, √ compared to Mincle KO, $ compared to Dectin-1 KO, # compared to Dectin-2 KO, € compared to MincleDectin-2 DKO2 (B & D) ROS (DHR-123) was measured by image flow cytometry (Amnis Imagestream^*x*^ MkII) in inflammatory monocytes/macrophages (B) and neutrophils (D) that interacted with labelled *C*. *albicans*. Representative images from 3 independent experiments are shown. BRF = Brightfield. Merged lane consists of CD11b, *C*. *albicans* and DHR-123. Ly6G is not included in the merged lane. (E-H) WT and CLR KO mice were injected with BIOgel i.p. and inflammatory cells were recovered by peritoneal lavage after 16–18 h and Ly6G^-^CD11b^+^ inflammatory monocytes/macrophages and Ly6G^+^CD11b^+^ neutrophils were purified. (E-F) Purified cell populations were incubated with luminol for 30 min and stimulated with *C*. *albicans*, opsonised *C*. *albicans* or PMA immediately prior to measurement of luminol luminescence for the shown timecourse. Graphs show cumulative data from 3 independent experiments. (1-way ANOVA with Bonferroni’s post-test on area under the curve). (G-H) Purified Ly6G^-^CD11b^+^ inflammatory monocytes/macrophages and Ly6G^+^CD11b^+^ neutrophils were incubated with *C*. *albicans*. Cells were lysed after 3 h and candidacidal activity was analysed by measuring CFU in cell lysates. Graphs display cumulative data from 3 independent experiments. (2-way ANOVA with Bonferroni’s post-test).

ROS production is considered important for protecting against *Candida* infections [42]. Therefore, we also examined the collective and contributing roles of Dectin-1, Dectin-2 and Mincle to *C*. *albicans*-induced ROS production by inflammatory monocytes/macrophages and neutrophils. We found that cells lacking Dectin-1, Dectin-2 or Mincle individually showed no major defects in *C*. *albicans*-induced ROS production by measuring Dihydrorhodamine 123 (DHR-123) fluorescence. However, inflammatory monocytes/macrophages lacking two or all three of these CLRs showed significant defects in *C*. *albicans*-induced ROS production (Fig 4A and 4B and S5 Fig). Dectin-1 KO neutrophils displayed a moderate reduction in *C*. *albicans*-induced ROS production however, additional loss of Mincle or Dectin-2 did not substantially increase this defect (Fig 4C and 4D and S5 Fig). As DHR-123 fluorescence only measures some reactive oxygen species (hydrogen peroxide, hypochlorous acid, peroxynitrite anion), we also purified the BIOgel-elicited inflammatory monocyte/macrophage and neutrophil populations from WT and Mincle-Dectin-2-Dectin-1 TKO1 mice to measure a broader spectrum of reactive oxygen species using a luminol assay. Using this assay, we did not observe any significant defects in ROS production by Mincle-Dectin-2-Dectin-1 TKO1 monocytes/macrophages (Fig 4E) or neutrophils (Fig 4F). We next measured the ability of WT and Mincle-Dectin-2-Dectin-1 TKO1 monocytes/macrophages and neutrophils to kill *C*. *albicans* and opsonised *C*. *albicans*. We did not observe any significant defect in the candidacidal ability of the multi-CLR KO cells (Fig 4G and 4H). These data indicate that while these CLRs minimally contribute to *C*. *albicans*-mediated induction of some specific reactive oxygen species, ROS production in general and *Candida* killing are largely independent of these CLRs.

### CLR collaboration mediates *C*. *albicans*-induced cytokine/chemokine production from inflammatory monocytes/macrophages

**Fig 5:**
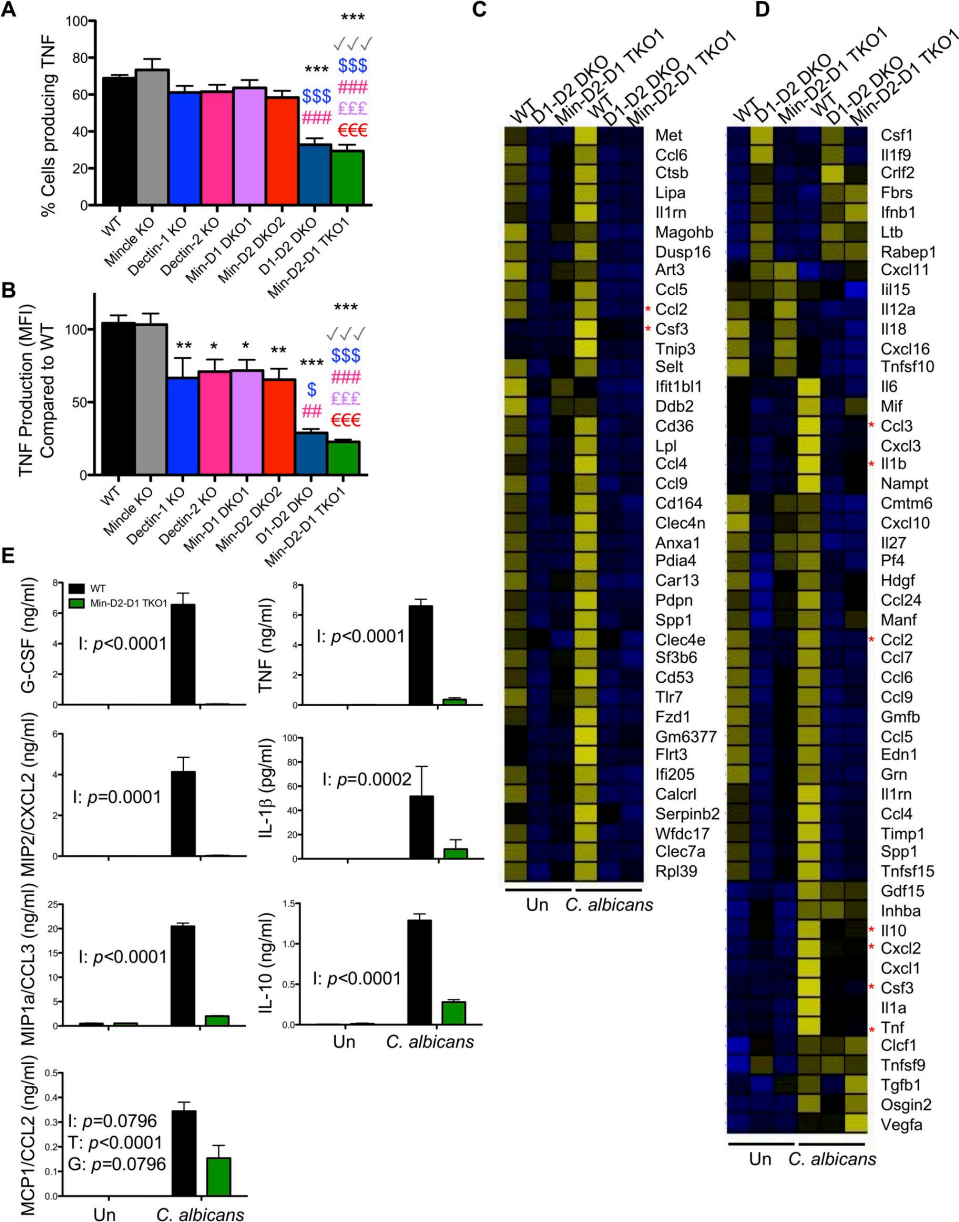
CLR collaboration mediates *C*. *albicans*-induced cytokine/chemokine production from inflammatory monocytes/macrophages. (A-D) WT and CLR KO mice were injected with BIOgel i.p. and inflammatory cells were recovered by peritoneal lavage after 16–18 h. (A-B) Cells were stimulated for 3 h with cell trace far red-labelled *C*. *albicans* at a 1:1 ratio in the presence of Brefeldin A. TNF levels were analysed by flow cytometry. Percentage Ly6G^-^CD11b^+^ inflammatory monocytes/macrophages that produced TNF—isotype (A) and MFI TNF production—isotype (B) was measured by flow cytometry in inflammatory monocytes/macrophages that interacted with labelled *C*. *albicans*. TNF MFI for WT cells interacting with *C*. *albicans* was set at 100%. (A-B) n = 2–7 mice. Graphs show cumulative data from 5 independent experiments. (1-way ANOVA with Bonferroni’s post-test). (C-D) Ly6C^hi^ inflammatory monocytes were purified by cell sorting. Cells were stimulated with *C*. *albicans* at a ratio of 3:1 (Cells:*Candida*) for 3 h. RNA was extracted and RNAseq analysis was performed. Data shows the mean from two replicates each of WT and Min-D2-D1 and one replicate of D1D2 DKO cells. 177 protein coding transcripts that showed 10 or more reads and an adjusted p value of <0.05 (Benjamin-Hochberg correction for multiple testing) were selected from the RNAseq data. Following Z transformation across genes, genes were clustered by K means of FPKM values into 3 clusters using Genesis software (S8 Fig). (C) Genes from Cluster 1 are displayed. (D) Heatmap displays genes with the GO terms chemokine activity (GO:0008009), cytokine activity (GO:0005125) and growth factor activity (GO:0008083) for transcripts that showed 10 or more reads. (C-D) Data range -2.5 (blue) to +2.5 (yellow). Asterisks highlight the genes chosen for validation. (E) Bone marrow monocytes were bead purified from WT and Min-D2-D1 TKO1 mice and stimulated with *C*. *albicans* at a ratio of 1:1 for 24 h in the presence of Amphotericin B. Cytokines and chemokines in the supernatants were measured by ELISA. Graphs display cumulative data from 4 independent experiments. I = Interaction, T = Treatment, G = Genotype (matched 2-way ANOVA on transformed data).

As cytokine production is important for the clearance of systemic *C*. *albicans* infections [43], we examined TNF production from monocytes/macrophages in response to *C*. *albicans*. Inflammatory myeloid cells recovered after BIOgel injection into WT and multi-CLR KO mice were incubated *ex vivo* with cell trace far red-labelled live *C*. *albicans* for 3 h. TNF production in the monocytes/macrophages that interacted with live *C*. *albicans* was measured by flow cytometry. Dectin-1 KO and Dectin-2 KO monocytes/macrophages displayed reduced *C*. *albicans*-induced TNF production, while Dectin-1-Dectin-2 DKO and Mincle-Dectin-2-Dectin-1 TKO1 cells displayed a more marked reduction in TNF production (Fig 5A and 5B and S7 Fig). The defect in TNF production appears to be independent of Mincle.

We next examined whether CLRs collaborated to more broadly influence inflammatory monocyte gene expression. Thus, we stimulated BIOgel-elicited monocytes isolated from WT, Dectin-1-Dectin-2 DKO and Mincle-Dectin-2-Dectin-1 TKO1 mice with *C*. *albicans* for 3 h and examined global gene expression using RNASeq analysis. We applied a filter to remove low expression transcripts (<10 reads) which revealed 177 significantly different (adjusted *p* value of <0.05) protein coding transcripts. Following Z transformation across genes, genes were clustered by K means into 3 clusters using Genesis software (S8 Fig). Cluster 1 consisted of genes that were higher basally and/or upregulated with *C*. *albicans* stimulation in WT cells compared to Dectin-1-Dectin-2 DKO and Mincle-Dectin-2-Dectin-1 TKO1 cells (Fig 5C). Ingenuity Pathway Analysis (IPA) revealed that these genes are important for the inflammatory response, cellular movement and cell-to-cell signalling. As many of the genes in this cluster were chemokines, we searched for the GO terms chemokine activity (GO:0008009), cytokine activity (GO:0005125) and growth factor activity (GO:0008083) for transcripts that showed 10 or more reads (Fig 5D). Many of these genes were present or induced in WT cells by *C*. *albicans* while they were absent or not induced in Dectin-1-Dectin-2 DKO and Mincle-Dectin-2-Dectin-1 TKO1 cells. The defect in the inflammatory response in Mincle-Dectin-2-Dectin-1 TKO1 monocytes was validated by ELISA measurement of chemokines and cytokines in cell supernatants 24 h after stimulation with *C*. *albicans* (Fig 5E). The ELISA results validated the findings in Fig 5C and 5D. These data indicate that Dectin-1 and Dectin-2 are vitally important for mediating *C*. *albicans*-induced chemokine and cytokine production from inflammatory monocytes.

### Dectin-1 is important for early neutrophil recruitment

**Fig 6:**
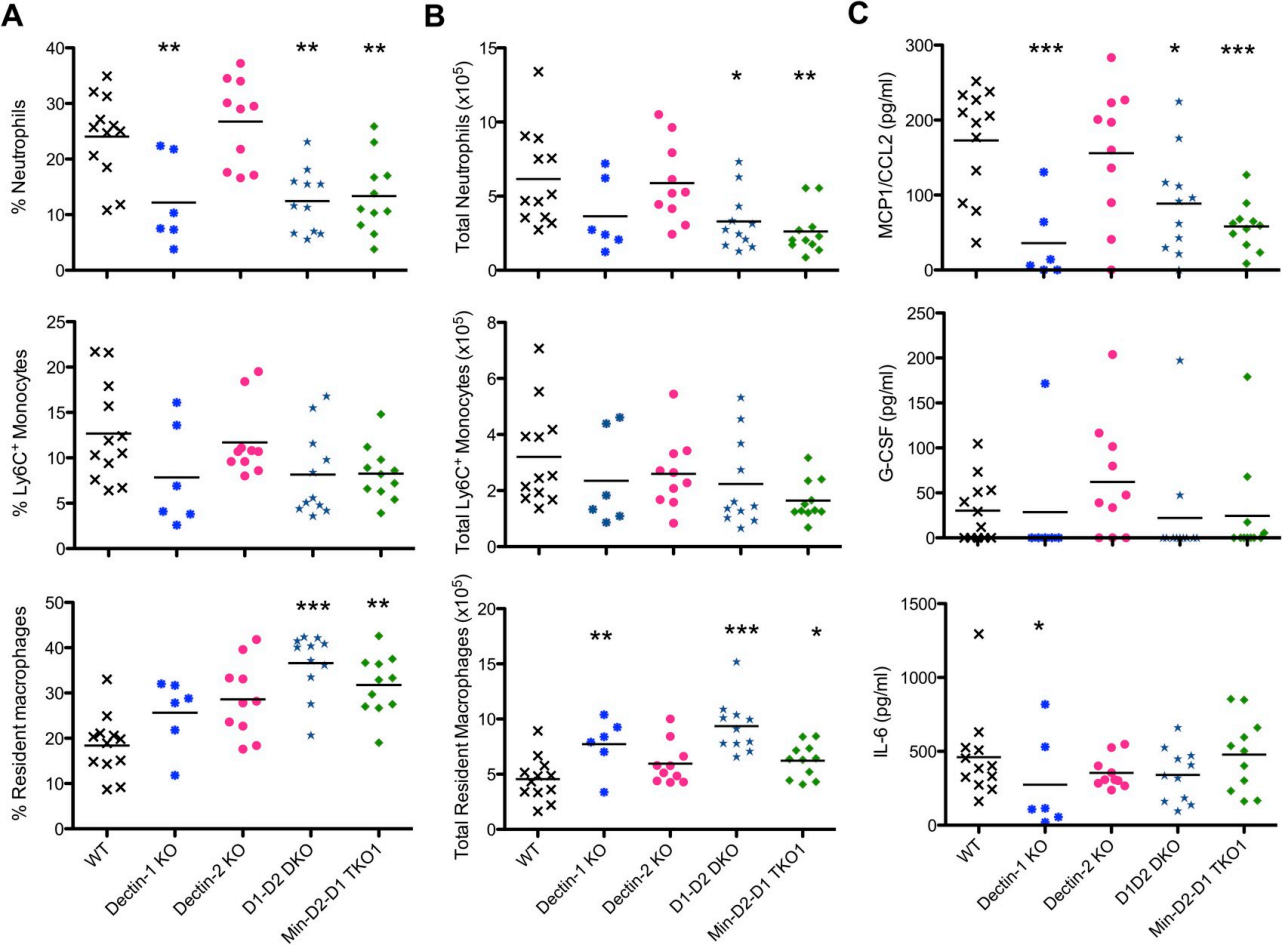
Dectin-1 is important for early neutrophil recruitment. **(**A-C) WT and CLR KO mice were injected with 1x10^5^ CFU *C*. *albicans* i.p. and inflammatory cells were recovered by peritoneal lavage after 4 h. (A-B) Percentage (A) and total number (B) of recruited neutrophils, Ly6^+^ monocytes and resident macrophages in the peritoneal lavages were measured by flow cytometry. (C) Chemokine and cytokine levels in the lavage fluid were measured by ELISA. (A-C) n = 6–12 mice. Graphs show cumulative data from 3 independent experiments. (1-way ANOVA with Bonferroni’s post-test–Performed on transformed data for total resident macrophages, G-CSF and IL-6; Kruskal-Wallis test with Dunn’s post-test for % Resident macrophages).

As *C*. *albicans* recognition and cytokine/chemokine production were highly defective in inflammatory monocytes and macrophages from multi-CLR KO mice, we hypothesised that early innate cell recruitment would be delayed in response to *C*. *albicans*. In addition, Taylor *et al* previously demonstrated that Dectin-1 KO mice displayed reduced inflammatory cell recruitment in response to *C*. *albicans* [14]. Using a peritoneal infection model, we therefore explored whether this defective inflammatory cell recruitment also occurred in the highly susceptible multi-CLR KO mice. We injected mice intraperitoneally with 1x10^5^ CFU *C*. *albicans* and 4 h later, we examined inflammatory cell recruitment to the peritoneum. In response to *C*. *albicans*, we observed reduced neutrophil recruitment to the peritoneum of Dectin-1 KO, Dectin-1-Dectin-2 DKO and Mincle-Dectin-2-Dectin-1 TKO1 but not Dectin-2 KO mice (Fig 6A and 6B). These same mice also demonstrated reduced resident macrophage emigration (Fig 6A and 6B) and reduced levels of Ccl2 in the lavage fluid. These data confirm that Dectin-1 is highly important for early neutrophil recruitment to the *C*.*albicans*-infected peritoneum.

### CLRs mediate the protective effect of inflammatory monocytes during infection with *C*. *albicans*

**Fig 7:**
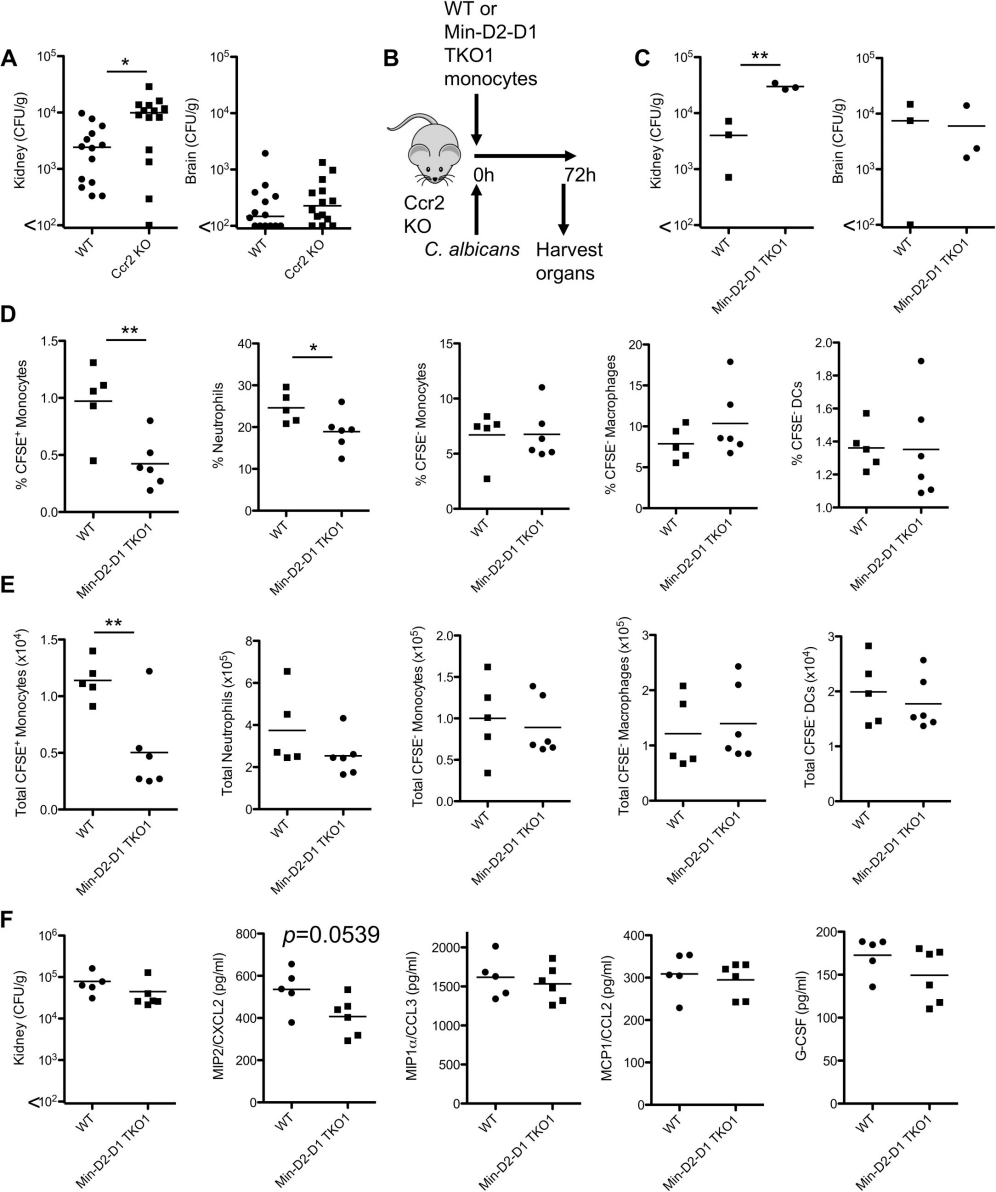
CLR collaboration is responsible for the protective effect of inflammatory monocytes during infection with *C*. *albicans*. (A) Quantification of fungal burden in the kidneys (left) or brains (right) of WT and Ccr2 KO mice 2 days after intravenous infection with 1x10^4^ CFU *C*. *albicans*. Graphs display cumulative data from 2 independent experiments. Each symbol represents one mouse. (Student’s *t* test on transformed data). (B) Protocol of WT or Min-D2-D1 TKO1 monocyte transfer and *C*. *albicans* infection in Ccr2 KO mice. (C) 3-4x10^6^ purified Ly6C^hi^ monocytes from WT or Min-D2-D1 TKO1 mice were adoptively transferred to Ccr2 KO mice followed by intravenous injection with 1.5x10^4^ CFU *C*. *albicans*. Organs were harvested after 72 h and fungal burden was determined. Graphs display cumulative data from 3 independent experiments. Each symbol represents one mouse. (Student’s paired *t* test). (D-F) 2.5x10^6^ purified monocytes from WT or Min-D2-D1 TKO1 mice were adoptively transferred to Ccr2 KO mice followed by intravenous injection with 1.5x10^4^ CFU *C*. *albicans*. (D-E) Kidneys were harvested after 24 h and myeloid cell recruitment to the kidneys was determined by flow cytometry. (F) Fungal burden and chemokine/cytokine production in the kidneys was determined. Graphs display cumulative data from 3 independent experiments. Each symbol represents one mouse. (Student’s *t* test).

As we identified highly defective cytokine/chemokine responses by multi-CLR KO monocytes *ex vivo* (Fig 5) and reduced chemokine production and neutrophil recruitment *in vivo* (Fig 6), we wanted to further examine the role of these CLRs in monocytes *in vivo*. Ccr2^+^ Ly6C^hi^ inflammatory monocytes have been proposed to be important during the first 48 h of systemic infection with *C*. *albicans* and Ccr2 KO mice are highly susceptible to systemic *C*. *albicans* infection [39, 40]. To confirm the protective effect of Ccr2^+^ Ly6C^hi^ monocytes in our facility during systemic infection with *C*. *albicans*, we infected WT and Ccr2 KO mice intravenously with 1x10^5^ CFU *C*. *albicans* SC5314. Similar to previous findings, we observed increased fungal burden in the kidneys of Ccr2 KO mice (Fig 7A). As inflammatory monocytes from Dectin-1-Dectin-2 DKO and Mincle-Dectin-2-Dectin-1 TKO1 mice display severely deficient responses (Figs 3–5) and these mice are highly susceptible to systemic *C*. *albicans* infection (Fig 1), we performed adoptive transfer experiments to determine whether these CLRs are responsible for the protective effect of monocytes during *C*. *albicans* infection (Fig 7B). Since Mincle was involved in recognition of *C*. *albicans* by inflammatory monocytes, we adoptively transferred Ly6C^hi^ monocytes from Mincle-Dectin-2-Dectin-1 TKO1 mice. We found that Ccr2 KO mice that received Ly6C^hi^ monocytes from WT mice displayed lower kidney fungal burden than mice that received Ly6C^hi^ monocytes from TKO1 mice, 72 h post-infection (Fig 7C). We then performed further adoptive transfer experiments to examine cell recruitment to the kidney, 24 h post-infection. We found significantly reduced numbers of adoptively transferred Mincle-Dectin-2-Dectin-1 TKO1 monocytes than WT monocytes in the kidneys of infected mice (Fig 7D and 7E). We also observed a subtle reduction in neutrophil recruitment to the kidneys (Fig 7D and 7E) and Cxcl2 production in the kidneys (Fig 7F) of mice who received Mincle-Dectin-2-Dectin-1 TKO1 monocytes compared to WT monocytes. These data indicate that these receptors mediate the protective effect of inflammatory monocytes during systemic infection with *C*. *albicans*.

## Discussion

Dectin-1, Dectin-2 and Mincle have been proposed to play various roles during anti-fungal immune responses, however how these receptors work together to respond to a systemic infection with *C*. *albicans* has not previously been determined. Here using novel multi-CLR KO mice, we identified individual, collaborative and redundant roles for Dectin-1, Dectin-2 and Mincle during systemic infection with *C*. *albicans*. We found that mice lacking two (Dectin-1 and Dectin-2) or three (Dectin-1, Dectin-2 and Mincle) CLRs exhibited dramatic susceptibility to systemic infection with *C*. *albicans* compared to WT or the other single or double KO mice strains. These multi-CLR KO mice displayed an excessive inflammatory response associated with uncontrolled fungal growth which likely resulted in immunopathology and organ failure. Surprisingly, all three CLRs were dispensable for neutrophil recognition/phagocytosis and killing of *C*. *albicans*. However, inflammatory monocytes/macrophages utilise all three CLRs for recognition/phagocytosis, but not for *Candida* killing. Furthermore, Dectin-1 and Dectin-2 are vitally important for the early *C*. *albicans*-induced cytokine and chemokine response from inflammatory monocytes, and Dectin-1 is required for early inflammatory cell recruitment likely mediated by resident macrophages. Our data indicates that these CLRs are important for mediating recognition and/or anti-fungal immune responses in inflammatory monocytes/macrophages in the following order: Dectin-1, Dectin-2, Mincle. We also found that the protective effect of inflammatory monocytes during systemic infection with *C*. *albicans* is mediated by these CLRs. Together these CLRs mediate recognition of *C*. *albicans* and the various immune responses required for clearance of C. *albicans* and therefore, maintain organ function during infection by controlling fungal growth.

Based on our data, we believe that Dectin-1 and Dectin-2 are important to mediate various anti-fungal responses by inflammatory monocytes/macrophages while they are largely dispensable for neutrophil anti-fungal responses. These data extend our knowledge of anti-fungal roles for Dectin-1 and Dectin-2 and we confirm that Mincle plays a subtle role in recognition of *C*. *albicans*. Interestingly, we found that *C*. *albicans*-induced recognition and phagocytosis is severely attenuated in inflammatory monocytes/macrophages from Mincle-Dectin-2-Dectin-1 TKO1 mice while these responses are mostly normal in neutrophils lacking these CLRs. Dectin-1 and Dectin-2 are expressed at higher levels on inflammatory monocytes/macrophages than on neutrophils which may help to explain the greater dependence on these CLRs by inflammatory monocytes/macrophages than neutrophils. In agreement with these data, Ferwerda *et al* showed that neutrophils from Dectin-1 deficient patients displayed normal phagocytosis of opsonised *C*. *albicans* [18]. However, it was also reported that murine Dectin-1 KO neutrophils displayed reduced recognition of non-opsonised zymosan and *C*. *albicans*, while recognition of opsonised particles was normal [41]. Similarly, an additional report showed that unopsonised *C*. *albicans* binding to human neutrophils could be partially blocked with the addition of soluble β-glucan or anti-Dectin-1, while the binding of opsonised *C*. *albicans* was not affected [44]. We did not exogenously opsonise the *C*. *albicans* in this study for the recognition assay, however it is possible that some opsonins were present in the cell preparations. In addition, the timing and ratio of cells:*Candida* differed between our experiments and the previously published data [41], which could potentially explain some of the discrepancies in our data. Another report found that CR3 was important for recognition of zymosan and ROS production by human neutrophils and that this was independent of Dectin-1 [45]. In addition, CR3 binds iC3b on opsonised *C*. *albicans* and it can also bind Pra1 and β-glucan on the cell surface/wall of *C*. *albicans* [46, 47], indicating that CR3 could potentially mediate neutrophil recognition of *C*. *albicans* and ROS production in the absence of these CLRs. However, as the inflammatory monocyte/macrophage population expresses higher levels of CD11b than neutrophils (S1 Fig), it is also possible that additional receptors expressed on neutrophils such as FcγR are involved in partially mediating these responses. While we have not determined here which receptors mediate neutrophil recognition of *C*. *albicans* it is clear from our data that Dectin-1, Dectin-2 and to a lesser extent Mincle mediate early recognition of *C*. *albicans* by inflammatory monocytes/macrophages.

In addition to reduced recognition of *C*. *albicans*, we hypothesised that the defective inflammatory monocyte response by TKO1 monocytes may affect neutrophil-mediated killing of *C*. *albicans*. A report recently showed that type I IFN from inflammatory monocytes induced IL-15 production which led to activation of NK cells and GM-CSF release to boost the candidacidal potential of neutrophils [39]. Furthermore, the neutrophils from a CARD9 deficient patient showed normal recognition and ROS production similar to our TKO1 neutrophils, however *Candida* killing was defective in the CARD9 deficient neutrophils [30]. However, we did not observe any defects in *Candida* killing in our TKO1 cells suggesting that the CARD9-dependent killing is mediated by different receptors than these CLRs for murine neutrophils, or that dependence on these CLRs for *Candida* killing differs between mouse and human neutrophils.

As neutrophil functions were largely normal, we then questioned why Mincle-Dectin-2-Dectin-1 TKO1 mice were so susceptible to systemic *Candida* infection. We hypothesised that the early innate response was severely defective in the susceptible multi-CLR KO mice. In fact it has previously been shown that the early inflammatory response including neutrophil recruitment was reduced in Dectin-1 KO mice compared to WT mice [14], and similarly, we found that neutrophil recruitment was also defective in the Dectin-1-Dectin-2 DKO and Mincle-Dectin-2-Dectin-1 TKO1 mice, 4 h after infection in a peritoneal model of *C*. *albicans* infection. This was likely due to the loss of Dectin-1 on resident macrophages resulting in defective activation of these cells [14]. Resident macrophages express Dectin-1 but not Dectin-2 [32], which fits with the normal neutrophil recruitment that we observed in Dectin-2 KO mice. In addition, our observation that the inflammatory response is largely absent in monocytes from the multi-CLR KO mice further indicates that the immune response to *C*. *albicans* during the first 24 h of infection is likely severely defective. We and others have shown that Ccr2 KO mice displayed increased susceptibility to systemic infection with *C*. *albicans* (Fig 7A) [39, 40]. Trafficking of Ly6C^hi^ inflammatory monocytes to infected organs including the kidney and spleen was severely reduced 24 h post-infection in Ccr2 KO mice. This was accompanied by a moderate reduction in the monocyte-derived DC population and reduced neutrophil recruitment to these organs [39]. These inflammatory monocytes were shown to be essential during the first 48 h post-infection to control fungal growth [40]. Here, we have shown that Dectin-1, Dectin-2 and Mincle mediate recognition and/or various anti-fungal responses of inflammatory monocytes and that these CLRs mediate the protective effect of inflammatory monocytes during systemic infection with *C*. *albicans*. Using an adoptive transfer model, we found reduced Mincle-Dectin-2-Dectin-1 TKO1 monocytes compared to WT monocytes in the kidneys of infected CCR2 KO mice. This could be due to reduced recruitment, survival or retention of these cells in the kidneys of infected mice. This was associated with a near significant reduction in Cxcl2 production in the kidney and a subtle reduction in neutrophil recruitment to the kidney of mice that received Mincle-Dectin-2-Dectin-1 TKO1 monocytes compared to WT monocytes. In line with this data, we observed defective chemokine production including neutrophil-recruiting chemokines by TKO1 inflammatory monocytes following incubation with *C*. *albicans* (Fig 5). Drummond *et al* recently showed that neutrophil recruitment to the brain during systemic infection with *C*. *albicans* was significantly impaired in Card9 KO mice due to reduced production of neutrophil-recruiting chemokines (Cxcl1, Cxcl2, Cxcl5) resulting in fungal invasion of the central nervous system [31]. These data indicate that these CLRs are vitally important for early innate chemokine/cytokine production and resulting inflammatory cell recruitment during the early stages of infection with *C*. *albicans*.

Due to the inability of Dectin-1-Dectin-2 DKO and Mincle-Dectin-2-Dectin-1 TKO1 mice to mount an appropriate early innate response to infection with *C*. *albicans*, these mice develop uncontrolled fungal growth particularly in the kidneys. In a model of invasive candidiasis, it has previously been shown that the spleen recruits large numbers of neutrophils and monocytes in response to the infection and successfully clears the pathogen from the spleen early during infection, which then results in a decline in myeloid cell numbers in the spleen. However, the kidney recruits much lower numbers of these myeloid cells and this correlates with increased fungal burden, persistent neutrophil accumulation and immunopathology in the kidney [48]. In our susceptible multi-CLR KO mice we observed a hyper-inflammatory response in the kidneys and spleen in association with the excessive fungal outgrowth in these mice. This inflammatory response did not resolve by 6 days post-infection. However, the inflammatory response in WT mice was much less pronounced and fungal burden was almost completely cleared in the WT mice at this time. We observed splenomegaly only in the highly susceptible Dectin-1-Dectin-2 DKO and Mincle-Dectin-2-Dectin-1 TKO1 mice, likely due to the excessive fungal growth in these mice. Patients who have survived candidemia while neutropenic can develop chronic disseminated candidiasis or hepatosplenic candidiasis. Following recovery of their neutrophil counts, these patients present with various symptoms including splenomegaly [49]. As mentioned above, in our highly susceptible multi-CLR KO mouse strains, we observed increased recruitment of myeloid cells, particularly monocytes and neutrophils, to the spleen and kidneys. In addition, the total number of T and NK cells and the total number of IFN-γ producing T/NK cells were increased the spleen of the multi-CLR KO mice. Furthermore, the slightly increased ability of multi-CLR KO splenocytes to produce IFN-γ following restimulation with *C*. *albicans* differs from previous reports with Dectin-1 KO, Dectin-2 KO or Card9 KO T cells [6, 7, 28], however these differences could be due to the use of live *C*. *albicans* in the presence of Amphotericin B versus heat killed *C*. *albicans* as this changes the ligand availability. The increased IFN-γ production is also likely due to the excessive fungal burden in these mice and these high levels of IFN-γ likely contribute to immunopathology in these mice. Taken together, these data indicate that failure to control fungal growth early during the infection due to defective early activation of monocytic cells and macrophages resulted in rapid excessive fungal outgrowth in Mincle-Dectin-2-Dectin-1 TKO1 mice, from which these mice were unable to recover.

In conclusion, we have shown that Dectin-1, Dectin-2 and to a lesser extent, Mincle mediate inflammatory monocyte/macrophage recognition of *C*. *albicans*. We have shown for the first time that the protective effect of inflammatory monocytes during systemic infection with *C*. *albicans* is mediated by these CLRs, while loss of these receptors did not have a direct effect on *C*. *albicans* recognition or ROS production by neutrophils. Loss of early control of *C*. *albicans* infection in the absence of these CLRs resulted in excessive fungal outgrowth and hyper-inflammation likely leading to multi-organ failure. The co-ordinated immune response through these CLRs to clear *C*. *albicans* infections could provide the basis for the design of novel immunotherapies for fungal or other pathogens that only naturally engage one of these receptors.

## Supporting information

S1 Table Differential splenocyte counts. Splenocytes were isolated from matched WT and multi-CLR KO mice. The stated markers were used to identify different cell populations by flow cytometry. Autofluorescence and F4/80 staining was used to identify red pulp macrophages while forward- and side-scatter profiles were used to define eosinophil and monocyte populations. Data represent cell numbers x10^6^ (mean ± s.e.m.). (n) = number of mice combined from 4 independent experiments. No significant differences were found with Kruskal-Wallis test with Dunn’s post-test.
(DOCX)

S1 Fig Characterisation of multi-CLR KO mice. (A) Schematic showing the Dectin-2 gene cluster (*Clec4n*, *Clec4d* and *Clec4e*) and Dectin-1 on mouse chromosome 6. Mincle KO mice contain a LoxP site in the *Clec4e* deleted allele. A LoxP site and three stop codons (one in each reading frame) were inserted into *Clec4n* gene (Dectin-2) in Mincle KO mice using Crispr-Cas9 technology to generate Mincle-Dectin-2 DKO2 mice. HA-L: Left Homology Arm, HA-R; Right Homology Arm. (B) BMDC from WT and Mincle-Dectin-2 DKO2 mice were lysed and immunoprecipitated with Isotype or α-Dectin-2, followed by immunoblotting with α-Dectin-2. Blot is representative of 3 independent experiments. (C-H) WT and CLR KO mice were injected with BIOgel i.p. and cells were recovered by peritoneal lavage after 16–18 h. Representative flow cytometry histograms showing (C-D) CLR surface expression on inflammatory monocyte/macrophage population (Ly6G^-^CD11b^+^) and (E-F) CLR surface expression on neutrophil population (Ly6G^+^CD11b^+^) from WT and multi-CLR KO. Plots are representative of 2–6 individual mice from 2–3 independent experiments. (G) MFI of receptor expression on inflammatory monocytes/macrophages or neutrophils. Graphs display cumulative data from 6 mice from 3 independent experiments. (Student’s *t* test). (H) Receptor surface expression on inflammatory monocyte/macrophage population from WT and multi-CLR KO. Plots are representative of 2–6 individual mice from 2–3 independent experiments.
(TIF)

S2 Fig Multi-CLR KO mice are dramatically susceptible to *C. albicans* infection. (A-B) Quantification of fungal burden in the kidneys (left) or brains (right) of WT and multi-CLR KO mice at time of humane end point or 30 days after intravenous infection with 5x10^4^ CFU (A) or 1.5x10^5^ CFU (B) *C*. *albicans*. Graphs display cumulative data from 7 (A) or 6 (B) independent experiments. Each symbol represents one mouse. (Kruskal-Wallis test with Dunn’s post-test on transformed data).
(TIF)

S3 Fig *C. albicans*-infected multi-CLR KO mice display enhanced inflammation in association with uncontrolled fungal burden. (A) Total myeloid cell numbers in the CD45^+^ cells from kidneys of WT and multi-CLR KO mice 20 h after intravenous infection with 1.5x10^4^ CFU *C*. *albicans* were quantified by flow cytometry. (B) Quantification of fungal burden in the kidneys of WT and multi-CLR KO mice 20 h after intravenous infection with 1.5x10^4^ CFU *C*. *albicans*. (B) Graphs display data from 1 experiment. Each symbol represents an individual mouse. (1-way ANOVA with Bonferroni’s post-test).
(TIF)

S4 Fig *C. albicans*-infected multi-CLR KO mice display enhanced inflammation in association with uncontrolled fungal burden. (A) Total myeloid cell populations and total splenocytes were quantified by flow cytometry (A) or Muse cell counter (B) in the spleens of WT and multi-CLR KO 4 days post-infection with 1.5x10^4^ CFU *C*. *albicans i*.*v*. (1-way ANOVA with Bonferroni’s post-test, Total splenocytes: Kruskal-Wallis test with Dunn’s post-test). (B-C) Total splenocytes were quantified 6 days (B) or 7 days (C) post-infection. (1-way ANOVA with Bonferroni’s post-test). (D) Total T and NK cell populations were quantified in the spleens 6 days post-infection. (E) 6 days post-infection splenocytes were restimulated with PMA and Ionomycin for 4 h in the presence of Brefeldin A. Total CD3^+^ T, CD4^+^ T, CD8^+^ T and NK cells producing IFN-γ were analysed by flow cytometry. (F) 6 days post-infection splenocytes were restimulated with live *C*. *albicans* for 48 h in the presence of Amphotericin B. IFN-γ and IL-17 levels in the supernatants were measured by ELISA. (D-F) Graphs display cumulative data from two independent experiments. Each symbol represents an individual mouse. (1-way ANOVA with Bonferroni’s post-test).
(TIF)

S5 Fig CLR collaboration mediates *C. albicans* recognition by inflammatory monocyte/macrophages but not neutrophils. (A-C) WT and CLR KO mice were injected with BIOgel i.p. and inflammatory cells were recovered by peritoneal lavage after 16–18 h. (A) Cells were stained with anti-Ly6G, anti-CD11b, anti-Ly6C and F4/80 and analysed by flow cytometry. (B-C) Cells were stimulated for 15 min with cell trace far red labelled *C*. *albicans* at a 1:1 ratio. Cells were loaded with the ROS indicator Dihydrorhodamine 123 (DHR-123) and stained with anti-Ly6G and anti-CD11b. (B-C) Representative flow plots displaying % Ly6G^-^CD11b^+^ inflammatory monocytes/macrophages (B) and Ly6G^+^CD11b^+^ neutrophils (C) that interacted with *C*. *albicans* are shown in the left of each panel. (B-C) Representative flow plots displaying ROS production (DHR-123) from inflammatory monocytes/macrophages (B) and neutrophils (C) that interacted with *C*. *albicans* (solid line) versus unstimulated cells (dashed line) are shown in the right of each panel. The geometric mean for ROS production is displayed in each flow plot.
(TIF)

S6 Fig CLR collaboration mediates *C. albicans* recognition by inflammatory monocytes/macrophages but not neutrophils. (A-B) WT and Min-D2-D1 TKO1 mice were injected with BIOgel i.p. and inflammatory cells were recovered by peritoneal lavage after 16–18 h. Cells were stained with anti-Ly6G and anti-CD11b. Cells were stimulated with cell trace far red-labelled *C*. *albicans* at a 1:1 ratio for 1.5 h and analysed by image flow cytometry (Amnis Imagestream^*x*^ MkII) for the course of the 1.5 h stimulation. Representative plots and images from 3 independent experiments are shown depicting internalised *C*. *albicans* or externally recognised *C*. *albicans* by inflammatory monocytes/macrophages (A) or neutrophils (B).
(TIF)

S7 Fig CLR collaboration mediates *C. albicans*-induced TNF production from inflammatory monocyte/macrophages. (A-B) WT and CLR KO mice were injected with BIOgel i.p. and inflammatory cells were recovered by peritoneal lavage after 16–18 h. Cells were stimulated for 3 h with cell trace far red-labelled *C*. *albicans* at a 1:1 ratio in the presence of Brefeldin A. TNF levels were analysed by flow cytometry. Representative flow plots displaying TNF production from Ly6G^-^CD11b^+^ inflammatory monocytes/macrophages that interacted with *C*. *albicans*.
(TIF)

S8 Fig CLR collaboration mediates *C. albicans*-induced cytokine/chemokine production from inflammatory monocytes/macrophages. Ly6C^hi^ inflammatory monocytes were purified by cell sorting. Cells were stimulated with *C*. *albicans* at a ratio of 3:1 (Cells:*Candida*) for 3 h. RNA was extracted and RNAseq analysis was performed. Data shows the mean from two replicates each of WT and Min-D2-D1 and one replicate of D1D2 DKO cells. 177 protein coding transcripts that showed 10 or more reads and an adjusted p value of <0.05 (Benjamin-Hochberg correction for multiple testing) were selected from the RNAseq data. Following Z transformation across genes, genes were clustered by K means of FPKM values into 3 clusters using Genesis^TM^ software. Data range -2.5 (blue) to +2.5 (yellow). The top molecular and cellular functions from Ingenuity Pathway Analysis for each cluster are displayed.
(TIF)
