# Supplementary material for: The protective effect of inflammatory monocytes during systemic C. albicans infection is dependent on collaboration between C-type lectin-like receptors
Source: PLoS Pathog. 2019 Jun 26;15(6):e1007850. doi: 10.1371/journal.ppat.1007850 (PMC6594653; doi:10.1371/journal.ppat.1007850)
Supplement: S1 Table — Splenocytes were isolated from matched WT and multi-CLR KO mice. The stated markers were used to identify different cell populations by flow cytometry. Autofluorescence and F4/80 staining was used to identify red pulp macrophages while forward- and side-scatter profiles were used to define eosinophil and monocyte populations. Data represent cell numbers x106 (mean ± s.e.m.). (n) = number of mice combined from 4 independent experiments. No significant differences were found with Kruskal-Wallis test with Dunn’s post-test. (DOCX) [file ppat.1007850.s001.docx]

| **Cell Type** | **Marker** | **WT (n)** | **Min-D1 DKO1 (n)** | **Min-D2 DKO2 (n)** | **D1-D2 DKO (n)** | **Min-D2-D1 TKO1 (n)** |
| --- | --- | --- | --- | --- | --- | --- |
| **Splenocytes** |  | 38.77±2.56 (10) | 52.62±4.00 (6) | 55.95±9.46 (6) | 37.23±3.54 (7) | 47.63±3.77 (7) |
| **B cells** | B220^+^ | 15.90±2.06 | 24.70±2.97 | 26.48±6.02 | 13.85±1.51 | 18.96±1.69 |
| **CD4^+^ T cells** | CD3^+^CD4^+^ | 6.33±1.00 | 8.88±0.71 | 9.53±1.11 | 3.76±0.81 | 6.08±1.28 |
| **CD8^+^ T cells** | CD3^+^CD8^+^ | 3.41±0.53 | 2.68±0.30 | 2.89±0.31 | 4.31±0.96 | 4.52±1.05 |
| **Natural Killer cells** | CD49b^+^CD3^-^ | 1.55±0.13 | 2.08±0.11 | 2.08±0.38 | 1.64±0.12 | 1.98±0.22 |
| **Dendritic cells** | CD11c^hi^MHCII^+^ | 0.42±0.03 | 0.51±0.04 | 0.57±0.11 | 0.41±0.04 | 0.57±0.06 |
| **Red Pulp Macrophages** | F4/80^hi^ | 0.63±0.05 | 0.84±0.09 | 0.83±0.25 | 0.87±0.18 | 0.84±0.11 |
| **Neutrophils** | Ly6G^hi^CD11b^+^ | 1.00±0.25 | 1.16±0.27 | 1.22±0.25 | 1.39±0.19 | 1.26±0.29 |
| **Eosinophils** | Ly6G^int^CD11b^+^ F4/80^+^ | 0.23±0.02 | 0.37±0.03 | 0.33±0.10 | 0.30±0.06 | 0.22±0.03 |
| **Monocytes** | Ly6G^lo/-^CD11b^+^ F4/80^+^ | 0.80±0.06 | 1.19±0.17 | 1.38±0.40 | 1.00±0.18 | 0.97±0.09 |

**S1 Table: Differential splenocyte counts.**
