# Supplementary figures and images for: The protective effect of inflammatory monocytes during systemic C. albicans infection is dependent on collaboration between C-type lectin-like receptors
Source: PLoS Pathog. 2019 Jun 26;15(6):e1007850. doi: 10.1371/journal.ppat.1007850 (PMC6594653; doi:10.1371/journal.ppat.1007850)

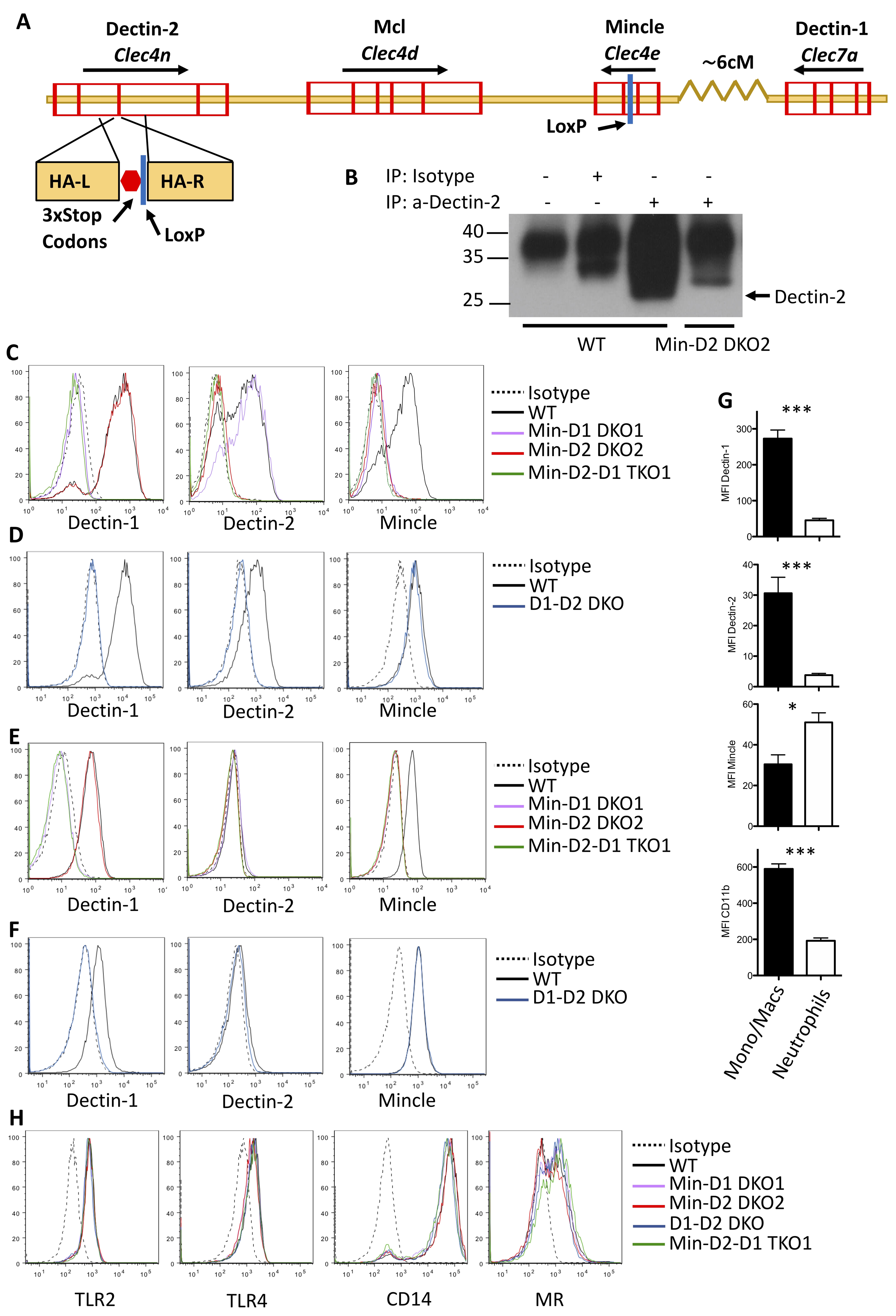

Supplement: S1 Fig — (A) Schematic showing the Dectin-2 gene cluster (Clec4n, Clec4d and Clec4e) and Dectin-1 on mouse chromosome 6. Mincle KO mice contain a LoxP site in the Clec4e deleted allele. A LoxP site and three stop codons (one in each reading frame) were inserted into Clec4n gene (Dectin-2) in Mincle KO mice using Crispr-Cas9 technology to generate Mincle-Dectin-2 DKO2 mice. HA-L: Left Homology Arm, HA-R; Right Homology Arm. (B) BMDC from WT and Mincle-Dectin-2 DKO2 mice were lysed and immunoprecipitated with Isotype or α-Dectin-2, followed by immunoblotting with α-Dectin-2. Blot is representative of 3 independent experiments. (C-H) WT and CLR KO mice were injected with BIOgel i.p. and cells were recovered by peritoneal lavage after 16–18 h. Representative flow cytometry histograms showing (C-D) CLR surface expression on inflammatory monocyte/macrophage population (Ly6G-CD11b+) and (E-F) CLR surface expression on neutrophil population (Ly6G+CD11b+) from WT and multi-CLR KO. Plots are representative of 2–6 individual mice from 2–3 independent experiments. (G) MFI of receptor expression on inflammatory monocytes/macrophages or neutrophils. Graphs display cumulative data from 6 mice from 3 independent experiments. (Student’s t test). (H) Receptor surface expression on inflammatory monocyte/macrophage population from WT and multi-CLR KO. Plots are representative of 2–6 individual mice from 2–3 independent experiments. (TIF) [file ppat.1007850.s002.tif]

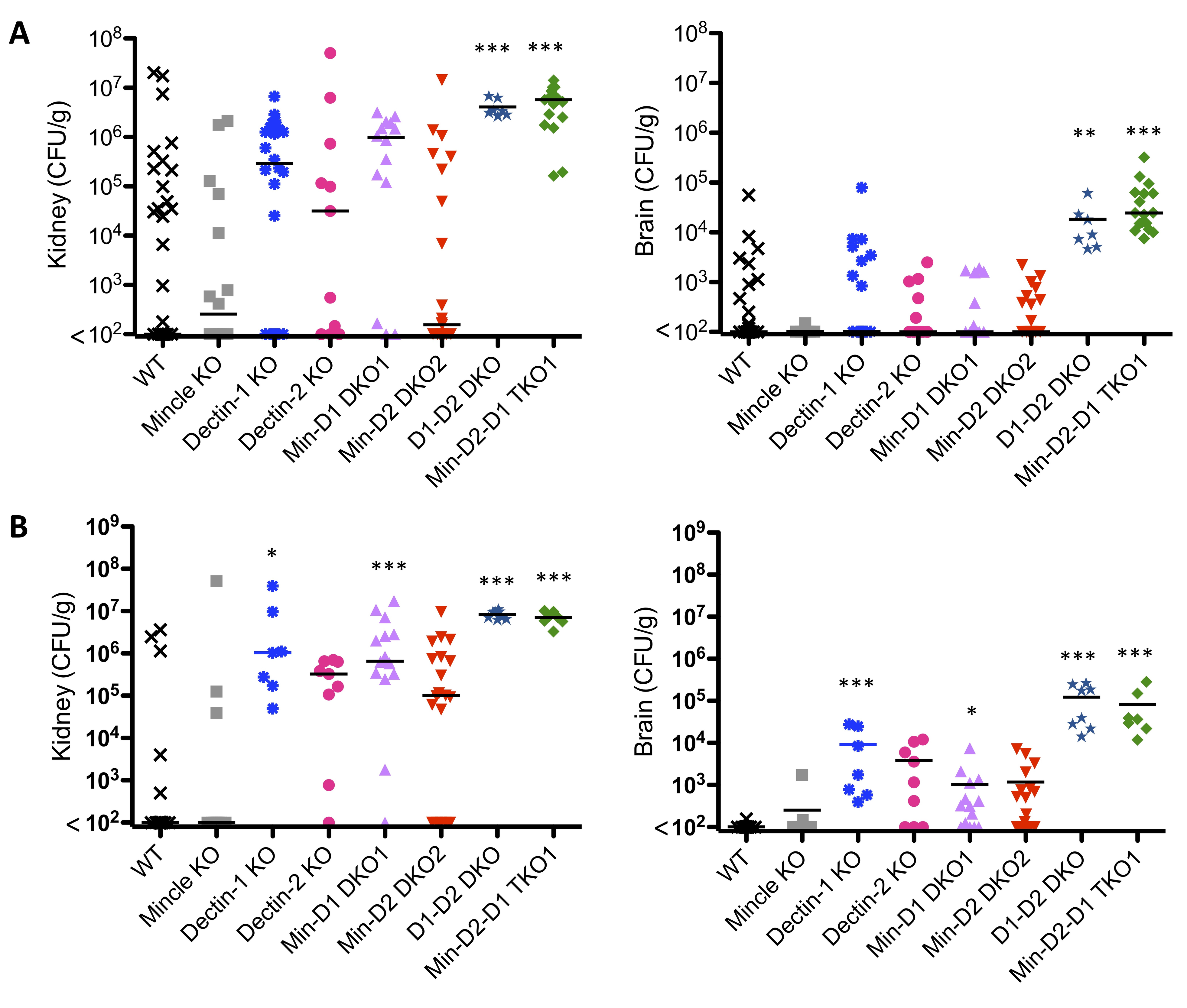

Supplement: S2 Fig — (A-B) Quantification of fungal burden in the kidneys (left) or brains (right) of WT and multi-CLR KO mice at time of humane end point or 30 days after intravenous infection with 5x10^4^ CFU (A) or 1.5x10^5^ CFU (B) C. albicans. Graphs display cumulative data from 7 (A) or 6 (B) independent experiments. Each symbol represents one mouse. (Kruskal-Wallis test with Dunn’s post-test on transformed data). (TIF) [file ppat.1007850.s003.tif]

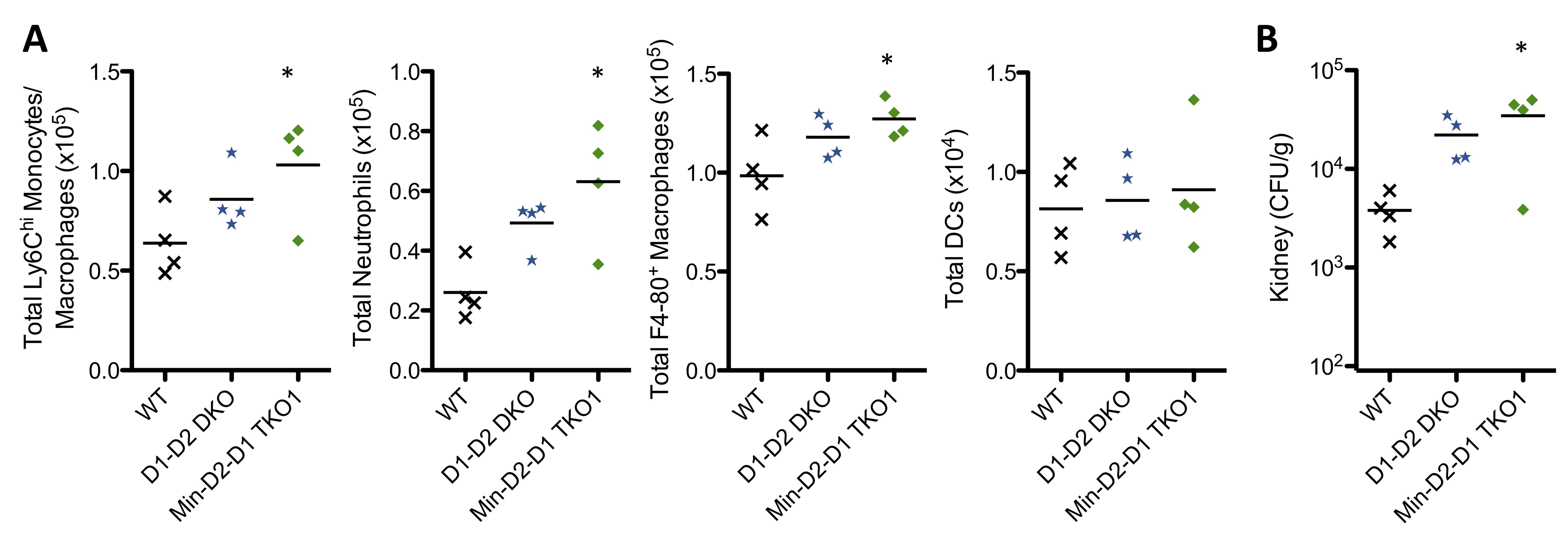

Supplement: S3 Fig — (A) Total myeloid cell numbers in the CD45+ cells from kidneys of WT and multi-CLR KO mice 20 h after intravenous infection with 1.5x10^4^ CFU C. albicans were quantified by flow cytometry. (B) Quantification of fungal burden in the kidneys of WT and multi-CLR KO mice 20 h after intravenous infection with 1.5x10^4^ CFU C. albicans. (B) Graphs display data from 1 experiment. Each symbol represents an individual mouse. (1-way ANOVA with Bonferroni’s post-test). (TIF) [file ppat.1007850.s004.tif]

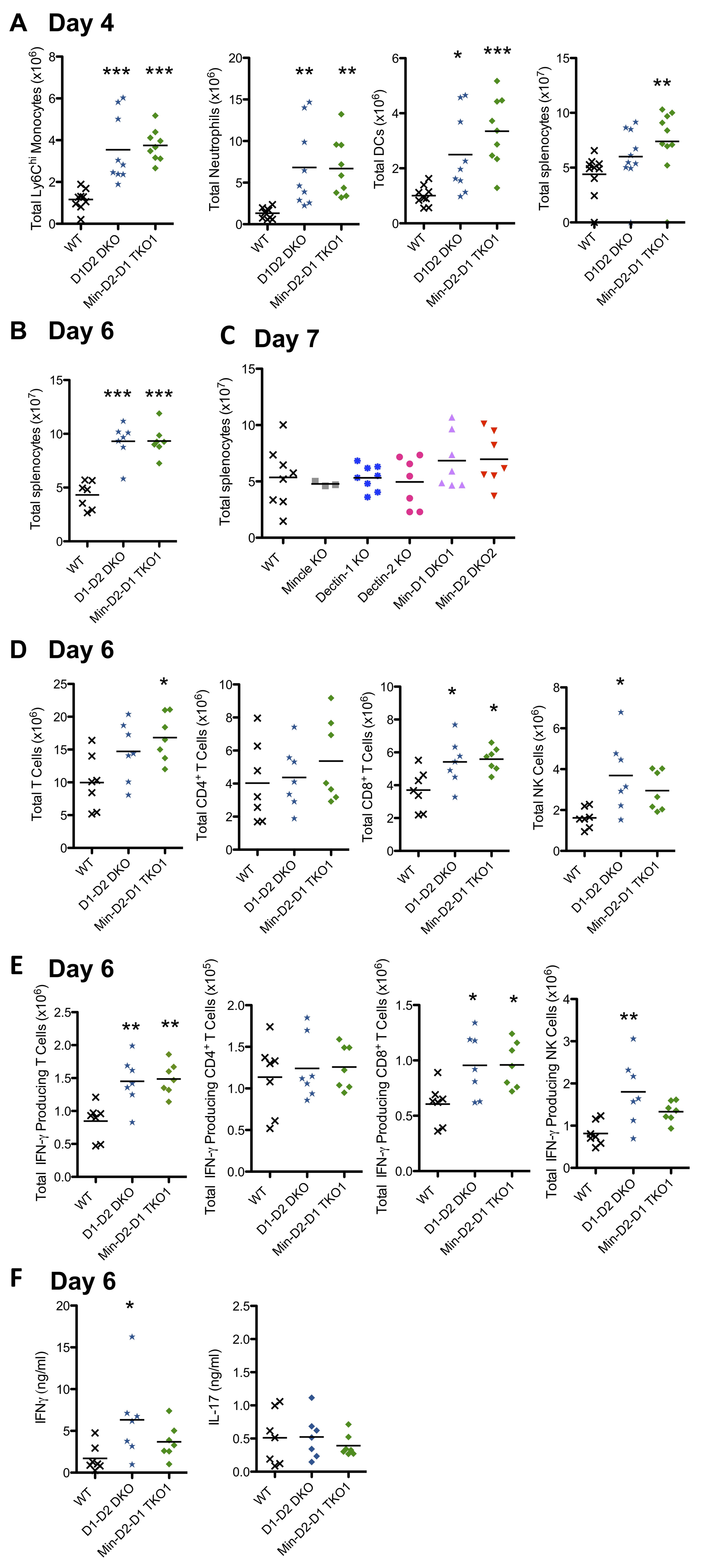

Supplement: S4 Fig — (A) Total myeloid cell populations and total splenocytes were quantified by flow cytometry (A) or Muse cell counter (B) in the spleens of WT and multi-CLR KO 4 days post-infection with 1.5x10^4^ CFU C. albicans i.v. (1-way ANOVA with Bonferroni’s post-test, Total splenocytes: Kruskal-Wallis test with Dunn’s post-test). (B-C) Total splenocytes were quantified 6 days (B) or 7 days (C) post-infection. (1-way ANOVA with Bonferroni’s post-test). (D) Total T and NK cell populations were quantified in the spleens 6 days post-infection. (E) 6 days post-infection splenocytes were restimulated with PMA and Ionomycin for 4 h in the presence of Brefeldin A. Total CD3+ T, CD4+ T, CD8+ T and NK cells producing IFN-γ were analysed by flow cytometry. (F) 6 days post-infection splenocytes were restimulated with live C. albicans for 48 h in the presence of Amphotericin B. IFN-γ and IL-17 levels in the supernatants were measured by ELISA. (D-F) Graphs display cumulative data from two independent experiments. Each symbol represents an individual mouse. (1-way ANOVA with Bonferroni’s post-test). (TIF) [file ppat.1007850.s005.tif]

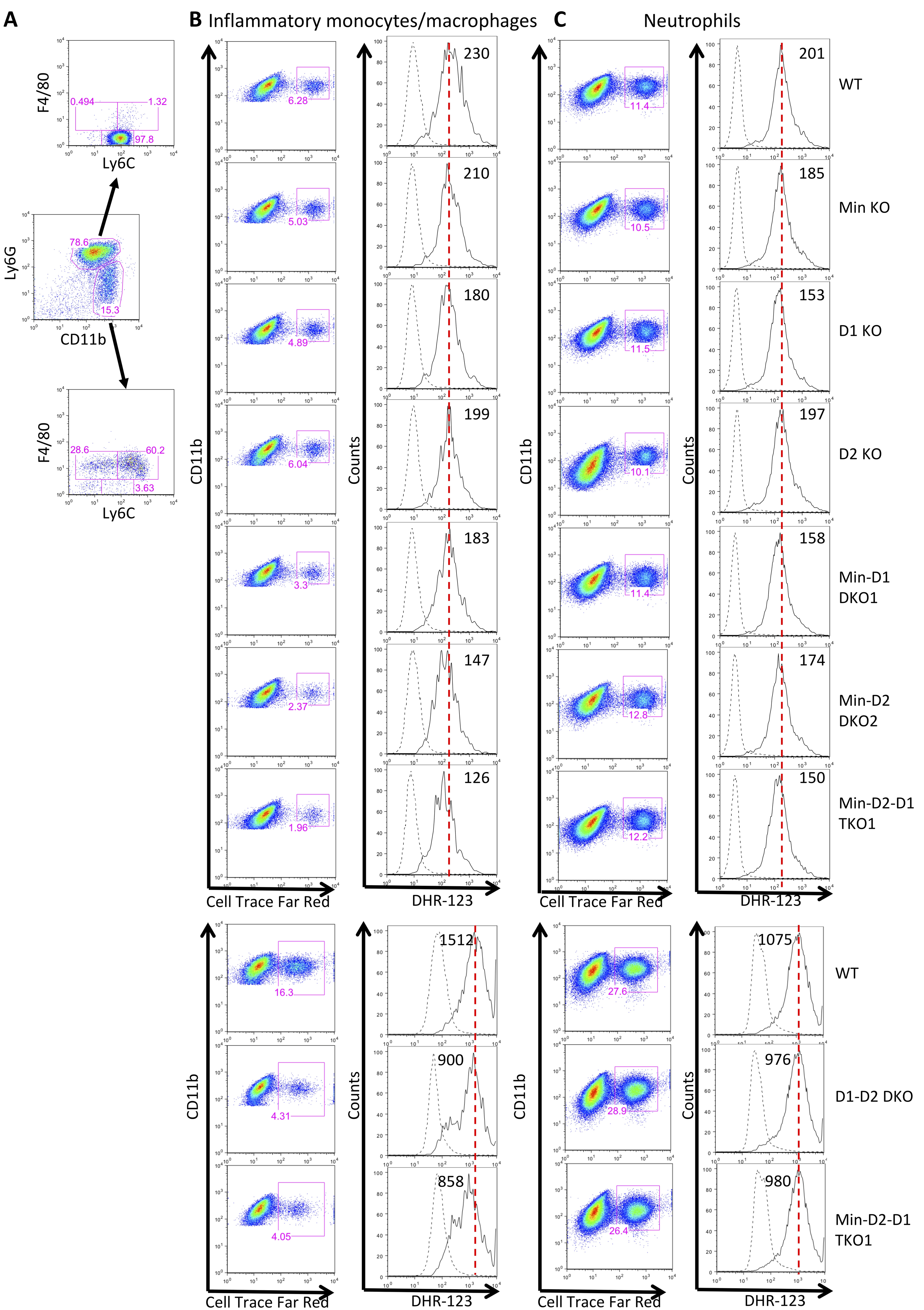

Supplement: S5 Fig — (A-C) WT and CLR KO mice were injected with BIOgel i.p. and inflammatory cells were recovered by peritoneal lavage after 16–18 h. (A) Cells were stained with anti-Ly6G, anti-CD11b, anti-Ly6C and F4/80 and analysed by flow cytometry. (B-C) Cells were stimulated for 15 min with cell trace far red labelled C. albicans at a 1:1 ratio. Cells were loaded with the ROS indicator Dihydrorhodamine 123 (DHR-123) and stained with anti-Ly6G and anti-CD11b. (B-C) Representative flow plots displaying % Ly6G-CD11b+ inflammatory monocytes/macrophages (B) and Ly6G+CD11b+ neutrophils (C) that interacted with C. albicans are shown in the left of each panel. (B-C) Representative flow plots displaying ROS production (DHR-123) from inflammatory monocytes/macrophages (B) and neutrophils (C) that interacted with C. albicans (solid line) versus unstimulated cells (dashed line) are shown in the right of each panel. The geometric mean for ROS production is displayed in each flow plot. (TIF) [file ppat.1007850.s006.tif]

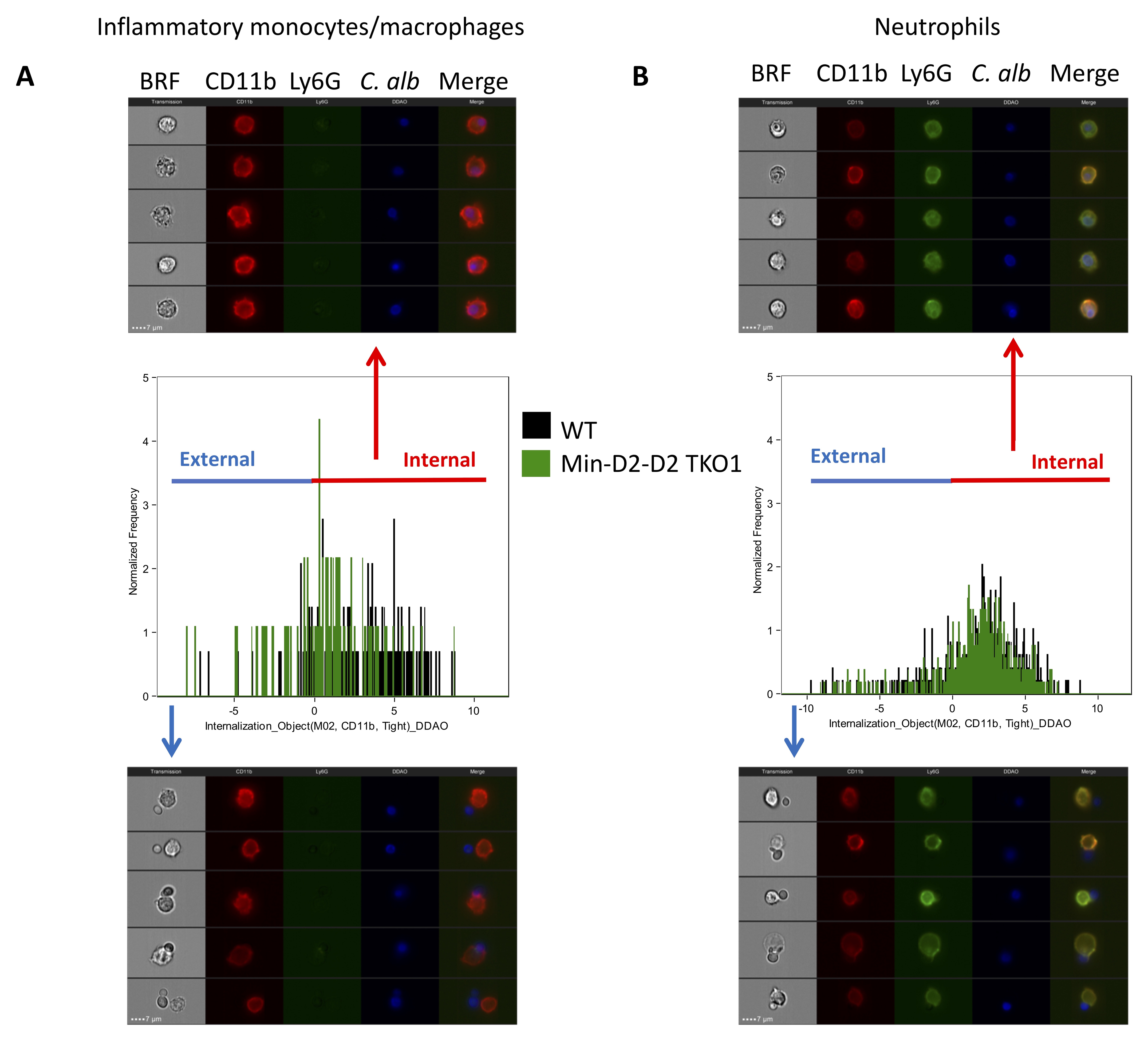

Supplement: S6 Fig — (A-B) WT and Min-D2-D1 TKO1 mice were injected with BIOgel i.p. and inflammatory cells were recovered by peritoneal lavage after 16–18 h. Cells were stained with anti-Ly6G and anti-CD11b. Cells were stimulated with cell trace far red-labelled C. albicans at a 1:1 ratio for 1.5 h and analysed by image flow cytometry (Amnis Imagestreamx MkII) for the course of the 1.5 h stimulation. Representative plots and images from 3 independent experiments are shown depicting internalised C. albicans or externally recognised C. albicans by inflammatory monocytes/macrophages (A) or neutrophils (B). (TIF) [file ppat.1007850.s007.tif]

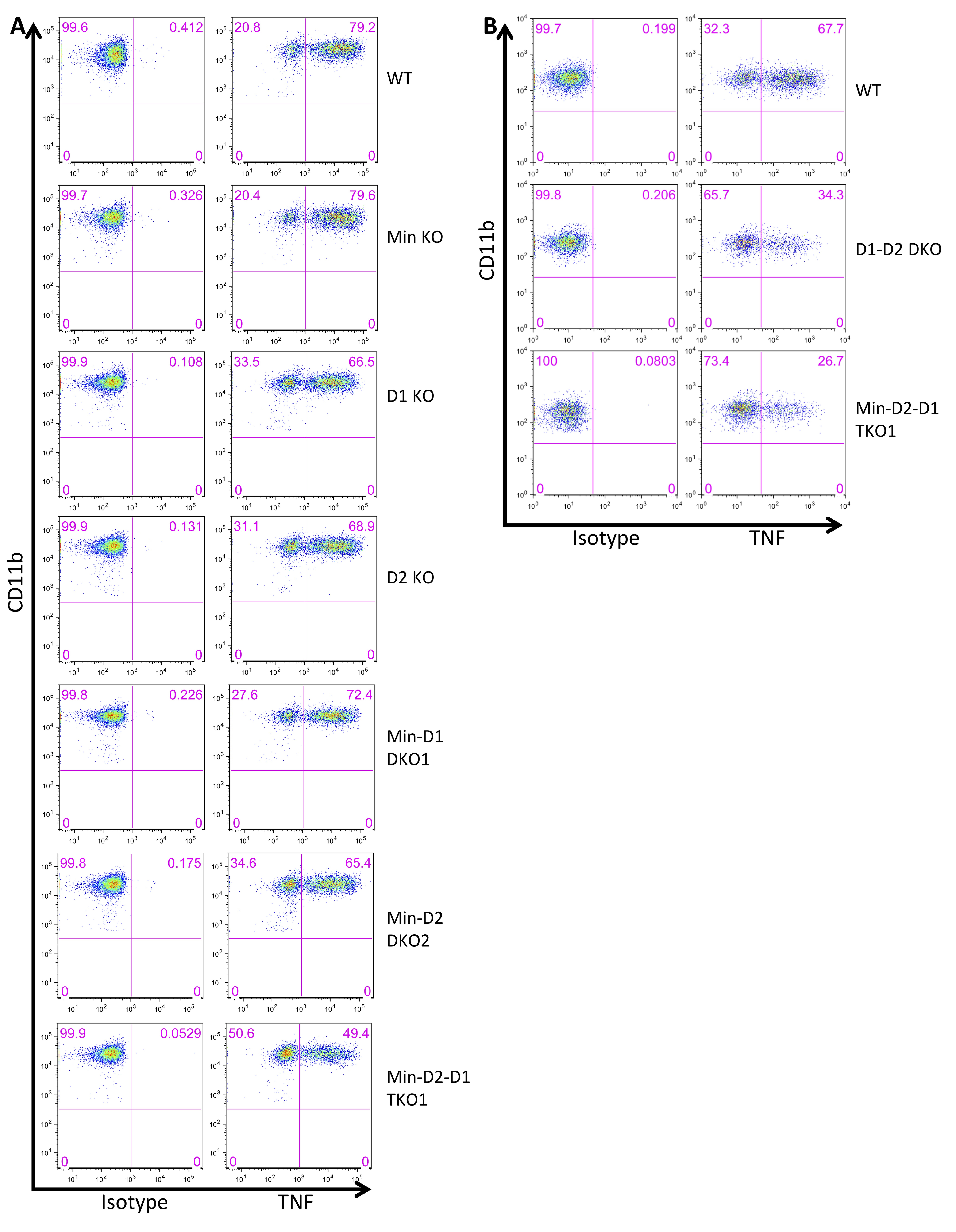

Supplement: S7 Fig — (A-B) WT and CLR KO mice were injected with BIOgel i.p. and inflammatory cells were recovered by peritoneal lavage after 16–18 h. Cells were stimulated for 3 h with cell trace far red-labelled C. albicans at a 1:1 ratio in the presence of Brefeldin A. TNF levels were analysed by flow cytometry. Representative flow plots displaying TNF production from Ly6G-CD11b+ inflammatory monocytes/macrophages that interacted with C. albicans. (TIF) [file ppat.1007850.s008.tif]

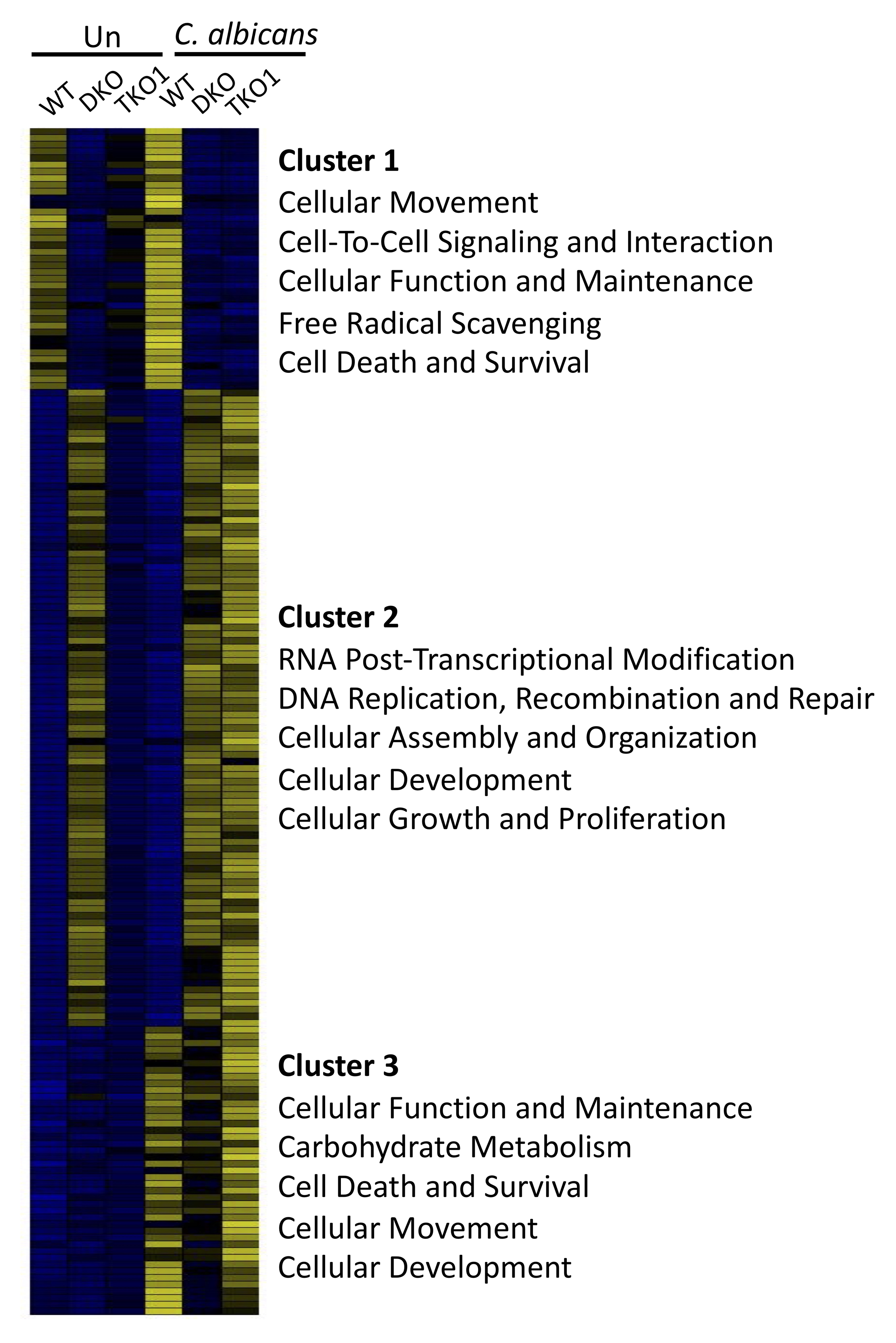

Supplement: S8 Fig — Ly6Chi inflammatory monocytes were purified by cell sorting. Cells were stimulated with C. albicans at a ratio of 3:1 (Cells:Candida) for 3 h. RNA was extracted and RNAseq analysis was performed. Data shows the mean from two replicates each of WT and Min-D2-D1 and one replicate of D1D2 DKO cells. 177 protein coding transcripts that showed 10 or more reads and an adjusted p value of <0.05 (Benjamin-Hochberg correction for multiple testing) were selected from the RNAseq data. Following Z transformation across genes, genes were clustered by K means of FPKM values into 3 clusters using GenesisTM software. Data range -2.5 (blue) to +2.5 (yellow). The top molecular and cellular functions from Ingenuity Pathway Analysis for each cluster are displayed. (TIF) [file ppat.1007850.s009.tif]
